# Data-driven causal analysis of observational biological time series

**DOI:** 10.7554/eLife.72518

**Published:** 2022-08-19

**Authors:** Alex Eric Yuan, Wenying Shou

**Affiliations:** 1 Molecular and Cellular Biology PhD program, University of Washington Seattle United States; 2 Basic Sciences Division, Fred Hutchinson Cancer Research Center Seattle United States; 3 Centre for Life’s Origins and Evolution, Department of Genetics, Evolution and Environment, University College London London United Kingdom; University of Zurich Switzerland; University of Zurich Switzerland

**Keywords:** time series, causality, model-free, surrogate data, convergent cross-mapping, Granger causality

## Abstract

Complex systems are challenging to understand, especially when they defy manipulative experiments for practical or ethical reasons. Several fields have developed parallel approaches to infer causal relations from observational time series. Yet, these methods are easy to misunderstand and often controversial. Here, we provide an accessible and critical review of three statistical causal discovery approaches (pairwise correlation, Granger causality, and state space reconstruction), using examples inspired by ecological processes. For each approach, we ask what it tests for, what causal statement it might imply, and when it could lead us astray. We devise new ways of visualizing key concepts, describe some novel pathologies of existing methods, and point out how so-called ‘model-free’ causality tests are not assumption-free. We hope that our synthesis will facilitate thoughtful application of methods, promote communication across different fields, and encourage explicit statements of assumptions. A video walkthrough is available (Video 1 or https://youtu.be/AlV0ttQrjK8).

## Introduction

Ecological communities perform important activities, from facilitating digestion in the human gut to driving biogeochemical cycles. Communities are often highly complex, with many species engaging in diverse interactions. To control communities, it helps to know causal relationships between variables (e.g. whether perturbing the abundance of one species might alter the abundance of another species). We can express these relationships either explicitly by proposing causal networks (Spirtes and Zhang, 2016; Chattopadhyay et al., 2019; Glymour et al., 2019; Runge et al., 2019b; Runge et al., 2019a; Sanchez-Romero et al., 2019; Leng et al., 2020), or implicitly by simply predicting the effects of new perturbations (Daniels and Nemenman, 2015; Mangan et al., 2016).

Ideally, biologists discover such causal relations from manipulative experiments. However, manipulative experiments can be infeasible or inappropriate: Natural ecosystems may not offer enough replicates for comprehensive manipulative experiments, and perturbations can be impractical at large scales and may have unanticipated negative consequences. On the other hand, there exists an ever-growing abundance of observational time series (i.e. without intentional perturbations). The goal of obtaining accurate causal predictions from these or similar data sets has motivated several complementary lines of investigation.

Determining causal relationships can become more straightforward if one already knows, or is willing to assume, a model that captures key aspects of the underlying process. For example, the Lotka-Volterra model popular in mathematical ecology assumes that species interact in a pairwise fashion, that the fitness effects from different interactions are additive, and that all pairwise interactions can be represented by a single equation form where parameters can vary to reflect signs and strengths of fitness effects. By fitting such a model to time series of species abundances and environmental factors, one can predict, for instance, which species interact or how a community might respond to certain perturbations (Stein et al., 2013; Fisher and Mehta, 2014; Bucci et al., 2016). However, the Lotka-Volterra equations often fail to describe complex ecosystems and chemically mediated interactions (Levine, 1976; Wootton, 2002; Momeni et al., 2017).

When our understanding is insufficient to support knowledge-based modeling, how might we formulate causal hypotheses? A large and rapidly growing literature attempts to infer causal relations from time series data without using a mechanistic model. Such methods are sometimes called ‘model-free’ (Coenen et al., 2020), although they typically rely on *statistical* models. Some of these methods avoid any equation-based description of the dynamics and instead examine some notion of ‘information flow’ between time series (Granger, 1980; Sugihara et al., 2012). Others deploy highly flexible equations that are not necessarily mechanistic (Granger, 1969; Barnett and Seth, 2014).

Here, we focus on three model-free approaches that have been commonly used to make causal claims in ecology research: pairwise correlation, Granger causality, and state space reconstruction. For each, we ask (1) what information does the method give us, (2) what causal statement might that information imply, and (3) when might the method lead us astray?

We found that answering these seemingly basic questions was at first surprisingly challenging for several reasons. First, modern causal discovery approaches have intellectual roots in several communities including philosophy, statistics, econometrics, and chaos theory, which sometimes use different words for the same idea, and the same word for different ideas. The word causality itself is a prime example: Many philosophers (and scientists) would say that X causes Y if an intervention upon X would result in a change in Y (Woodward, 2016; Pearl, 2000). Granger’s original works instead defined causality to be about how important the history of X is in predicting Y (Granger, 1969; Granger, 1980), and in the nonlinear dynamics field, causality is sometimes used to mean that the trajectories of X and Y have certain shared geometric or topological properties (Harnack et al., 2017). Such language, while unproblematic when confined to a single community, can nevertheless obscure important differences between methods from different communities. A second challenge is that in methodological articles, key assumptions are sometimes hidden in algorithmic details, or simply not mentioned. Finally, some methods deal with nuanced or advanced mathematical ideas that can be difficult even for those with quantitative training. Given these challenges, it is no surprise that efforts to infer causal relationships from observational time series have sometimes been highly controversial, with an abundance of ‘letters to the editor’, sometimes followed by impassioned dialogue (Luo et al., 2015; Baskerville and Cobey, 2017; Tiokhin and Hruschka, 2017; Schaller et al., 2017; Barnett et al., 2018).

We have tried to balance precision and readability in this review. To accomplish this, we devised new ways to visualize key concepts. We also compare all methods to a common definition of causality that is useful to experimental scientists. We provide refreshers and discussions of mathematical notions in the Appendices. Lastly, a video walkthrough covering many of the key concepts and takeaway messages is available at https://youtu.be/AlV0ttQrjK8; and as Video 1. Our goals are to inform, to facilitate communication across different fields, and to encourage explicit statements of methodological assumptions and caveats. For a broad overview of time series causal methods in Earth sciences or more technical reviews, see Runge et al., 2019b and Peters et al., 2017; Runge, 2018b respectively.

**Video 1.** Video walkthrough.

## Dependence, correlation, and causality

### Causality

**Figure 1.**
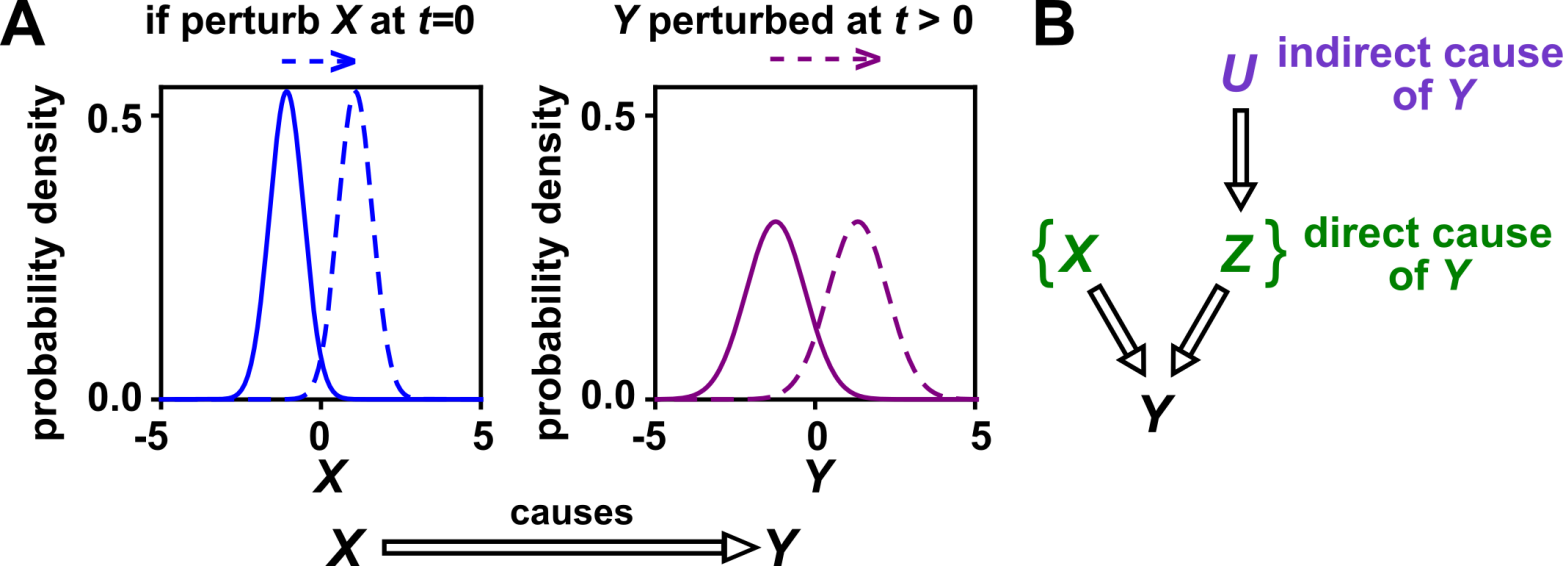
Causality. (**A**) Definition. If a perturbation in X can result in a change in future values of Y, then X causes Y. This definition does not require that *any* perturbation in X will perturb Y. For example, if the effect of X on Y has saturated, then a further increase in X will not affect Y. In this article, causality is represented by a hollow arrow. To embody probabilistic thinking (e.g. drunk driving increases the chance of car accidents; Pearl, 2000), X and Y are depicted as histograms. Sometimes, perturbations in one variable can change the current value of another variable if, for example, the two variables are linked by a conservation law (e.g. conservation of energy). Some have argued that these are also causal relationships (Woodward, 2016). (**B**) Direct versus indirect causality. The direct causers of Y are given by the minimal set of variables such that once the entire set is fixed, no other variables can cause Y. Here, three variables X, Z, and U activate Y. The set {X,Z} constitutes the direct causers of Y (or Y’s ‘parents‘ [Hausman and Woodward, 1999; Pearl, 2000]), since if we fix both X and Z, then Y becomes independent of U. If a causer is not direct, we say that it is indirect. Whether a causer is direct or indirect can depend on the scope of included variables. For example, suppose that yeast releases acetate, and acetate inhibits the growth of bacteria. If acetate is not in our scope, then yeast density has a direct causal effect on bacterial density. Conversely, if acetate is included in our scope, then acetate (but not yeast) is the direct causer of bacterial density (since fixing acetate concentration would fix bacterial growth regardless of yeast density). When we draw interaction networks with more than two variables, hollow arrows between variables denote direct causation.

In this article, we use the definition of ‘causality’ that is common in statistics and intuitive to scientists: X has a causal effect on Y (‘X causes Y’ or ‘X is a causer; Y is a causee’ or ‘X is a cause; Y is an effect’) if some externally applied perturbation of X can result in a perturbation in Y (Figure 1A). We say that X and Y are *causally related* if X causes Y, Y causes X, or some other variable causes both. Otherwise, X and Y are *causally unrelated*. Additionally, one can talk about direct versus indirect causality (Figure 1B; see legend for definitions). A surprising result from past several decades of causality research is that there are in fact some conditions under which directional causal structures can be correctly inferred (‘identified’) from purely observational data (Spirtes and Zhang, 2016; Peters et al., 2017; Hitchcock, 2020b) (e.g. Appendix 2—figure 2, last row). However, empirical time series often do not contain enough information for easy causal identifiability (Spirtes and Zhang, 2016; Glymour et al., 2019).

### Correlation versus dependence

The adage ‘correlation is not causality’ is well-known to the point of being cliché (Sugihara et al., 2012; Coenen and Weitz, 2018; Carr et al., 2019; Mainali et al., 2019). Yet, to dismiss correlative evidence altogether seems too extreme. To make use of correlative evidence without being reckless, it helps to distinguish between the terms ‘correlation’ and ‘dependence’. When applied to ecological time series, the term ‘correlation’ is often used to describe some statistic that quantifies the similarity between two observed time series (Weiss et al., 2016; Coenen and Weitz, 2018). Examples include Pearson’s correlation coefficient and local similarity (Ruan et al., 2006). In contrast, statistical dependence is a hypothesis about the probability distributions that produced those time series, and has close connections to causality.

Dependence has a precise definition in statistics, and is most easily described for two binary events. For instance, if the incidence of vision loss is higher among diabetics than among the general population, then vision loss and diabetes are statistically dependent. In general, events A and B are dependent if across many independent trials (e.g. patients), the probability that A occurs given that B has occurred (e.g. incidence of vision loss among diabetics only) is different from the background probability that A occurs (e.g. background incidence of vision loss). If A and B are not dependent, then they are called independent. The concept of dependence is readily generalized from binary events to numerical variables, and also to vectors such as time series (Appendix 1).

Dependence is connected to causation by the widely accepted ‘Common Cause Principle’: *if two variables are dependent, then they are causally related* (i.e. one causes the other, or both share a common cause; Peters et al., 2017; Runge et al., 2019a; Hitchcock, 2020b; Hitchcock and Rédei, 2020a). Note however that if one mistakenly introduces selection bias, then two independent variables can appear to be dependent (Appendix 2—figure 3). The closely related property of conditional dependence (i.e. whether two variables are dependent after statistically controlling for certain other variables; Appendix 1) can be even more causally informative. In fact, when conditional dependence (and conditional independence) relationships are known, it is sometimes possible to infer most or all of the direct causal relationships at play, even without manipulative experiments or temporal information. Many of the algorithms that accomplish this rely on two technical but often reasonable assumptions: the ‘causal Markov condition’, which allows one to infer causal information from conditional *dependence*, and the ‘causal faithfulness condition’, which allows one to infer causal information from conditional *independence* (Appendix 2; Peters et al., 2017; Glymour et al., 2019; Hitchcock, 2020b).

In sum, whereas a correlation is a statistical description of data, statistical dependence is a hypothesis about the relationship between the underlying probability distributions. Dependence is in turn linked to causality. Below, we discuss tests that use correlation to detect dependence in time series.

### Testing for dependence between time series using surrogate data

Despite its scientific usefulness, dependence between time series can be treacherous to test for. This is because time series are often autocorrelated (e.g. what occurs today influences what occurs tomorrow), so that a single pair of time series contains information from only a single trial. If one has many trials that are independent and free of systematic differences (e.g. ≥20 as in some laboratory microcosm experiments), the task is relatively easy: One can test whether the abundances of species X and Y are statistically dependent by comparing the correlation between X and Y abundance series from the same trial with those between X and Y abundance series from different trials (Appendix 1—figure 4; see also Moulder et al., 2018). However, a large trial number is generally a luxury and often only one trial is available. In such cases, attempting to discern whether two time series are statistically dependent is like attempting to divine whether diabetes and vision loss are dependent with only a single patient (i.e. we have an ‘n-of-one problem’). As one possible remedy, there are parametric tests using the Pearson correlation coefficient that account for autocorrelation. In these tests, one estimates the correlation coefficient between time series, and evaluates its statistical significance using the variance of the null distribution (Afyouni et al., 2019). However, the calculation of this variance relies on estimates of the autocorrelation at each lag for both time series, which can be highly uncertain (Pyper and Peterman, 1998; Ebisuzaki, 1997). Furthermore, after estimating the variance, one must also assume the shape of the null distribution before a p-value can be assigned to the correlation.

Alternatively, the n-of-one problem is often addressed by a technique called surrogate data testing. Specifically, one computes some measure of correlation between two time series X and Y. Next, one uses a computer to simulate replicates of Y that might have been obtained if X and Y were independent (see below). Each simulated replicate is called a ‘surrogate’ Y. Finally, one computes the correlation between X and each surrogate Y. A p-value (representing evidence against the null hypothesis that X and Y are independent) is then determined by counting how many of the surrogate Ys produce a correlation at least as strong as the real Y. For example, if we produced 19 surrogates and found the real correlation to be stronger than all 19 surrogate correlations, then we would write down a p-value of 1/(1+19)=0.05. Ideally, if two time series are independent, then we should register a a p-value of 0.05 (or less) in only 5% of cases.

**Figure 2.**
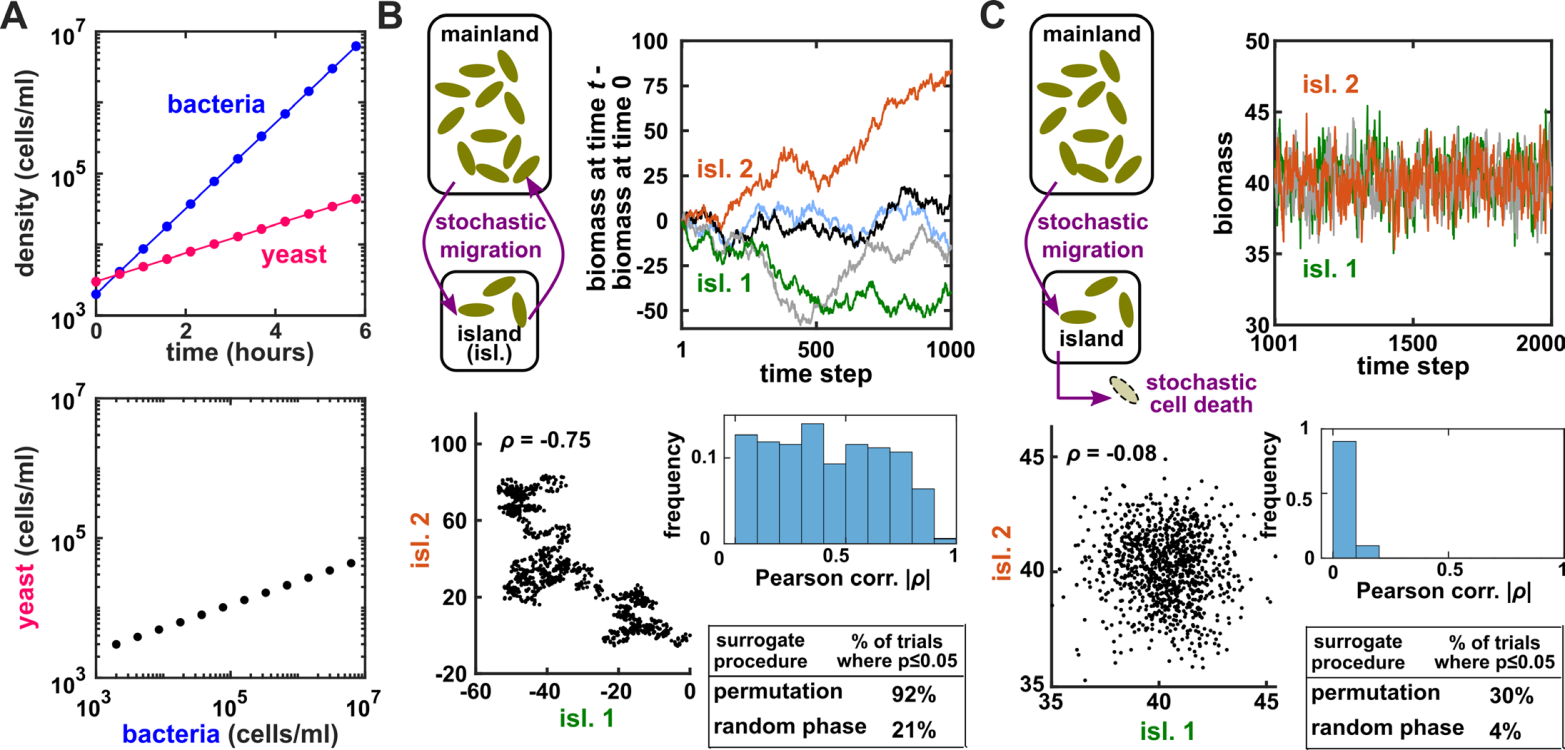
Two independent temporal processes can appear significantly correlated when compared to an inappropriate null model. (**A**) Densities of independent yeast and bacteria cultures growing exponentially are correlated. (**B, C**) Correlation between time series of two independent island populations can appear significant if inappropriate tests are used. (**B**) In an island (“isl”), individuals stochastically migrate to and from the mainland in roughly equal numbers so that total island biomass follows a random walk. At each time step, the net change in island biomass is drawn from a standard normal distribution (mean = 0; standard deviation = 1 biomass unit). (**C**) An island population receives cells through migration and loses cells via death. Observations are made after 1000 steps, so that the population size has reached an equilibrium. For both (**B**) and (**C**), we performed 1000 simulations in which we calculated the Pearson correlation coefficient of a pair of independent islands populations. Both panels contain: example time series (upper right), a scatterplot comparing two independent islands (lower left), the distribution of Pearson correlation coefficient strength (blue shading), and the proportion of simulations in which the correlation was deemed significant (p≤0.05) by surrogate data tests using either permutation or phase randomization (see main text). Ideally, the proportion of correlations that are significant (false positives) should not exceed 5%. The strength of correlation is weaker in (**C**) compared to (**B**), yet still often significant according to the permutation test. See Appendix 5 for more details.

Several procedures can be used to produce surrogate time series, each corresponding to an assumption about how the original time series was generated (Lancaster et al., 2018). One popular procedure is to simply shuffle the values of a time series (Ruan et al., 2006; Eiler et al., 2012; Shade et al., 2013; Cyriaque et al., 2020). This procedure, often called permutation, assumes that all possible orderings of the time points in the series are equally likely. This assumption is commonly violated in time series due to autocorrelation, and thus the test is often invalid. For example, for independent time series in Figure 2B–C, this test returns p<0.05 at rates of 30∼92%, much higher than 5%. Nevertheless, permutation testing has appeared in many applied works, perhaps because it has been the default option in some popular software packages. Another procedure for generating surrogates is called phase randomization. It first uses the Fourier transform to represent a time series as a sum of sine waves, then randomly shifts each of the component sine waves in time, and finally sums the phase-shifted components (Ebisuzaki, 1997; Schreiber and Schmitz, 2000; Andrzejak et al., 2003; Appendix 3—figure 1). This procedure is considered appropriate when the original time series is obtained from a linear, Gaussian, and stationary process (Andrzejak et al., 2003; Lancaster et al., 2018), where ‘linear’ means that future values depend linearly on past values, ‘Gaussian’ means that any subsequence follows a multivariate Gaussian distribution, and ‘stationary’ means that this distribution does not change over time. See Chan, 1997 for a discussion of exact requirements. Indeed, this test performed well (with a false positive rate of 4%) when time series satisfied its assumptions (Figure 2C), and poorly when the stationarity assumption was violated (with a false positive rate of 21%; Figure 2B). Other surrogate data procedures include time shifting (Andrzejak et al., 2003), the block bootstrap (Papana et al., 2017), and the twin method (Thiel et al., 2006). Some surrogate data tests have been shown to perform reasonably well even when the exact theoretical requirements are unmet or unknown (Thiel et al., 2006; Papana et al., 2017), but a more comprehensive benchmarking effort is needed to map out each method’s valid domain in practice.

In sum, surrogate data allow a researcher to use an observed correlation statistic to test for dependence under some assumption about the data-generating process. Dependence indicates the presence of a causal relationship, and conditional dependence can sometimes even indicate the direction (Hitchcock, 2020b; Glymour et al., 2019; Heinze-Deml et al., 2018; Appendix 2—figure 2). Below we consider Granger causality and state space reconstruction, two approaches that can be used to directly infer the direction of causality from time series.

## Granger causality: intuition, pitfalls, and implementations

### Intuition and formal definitions

**Figure 3.**
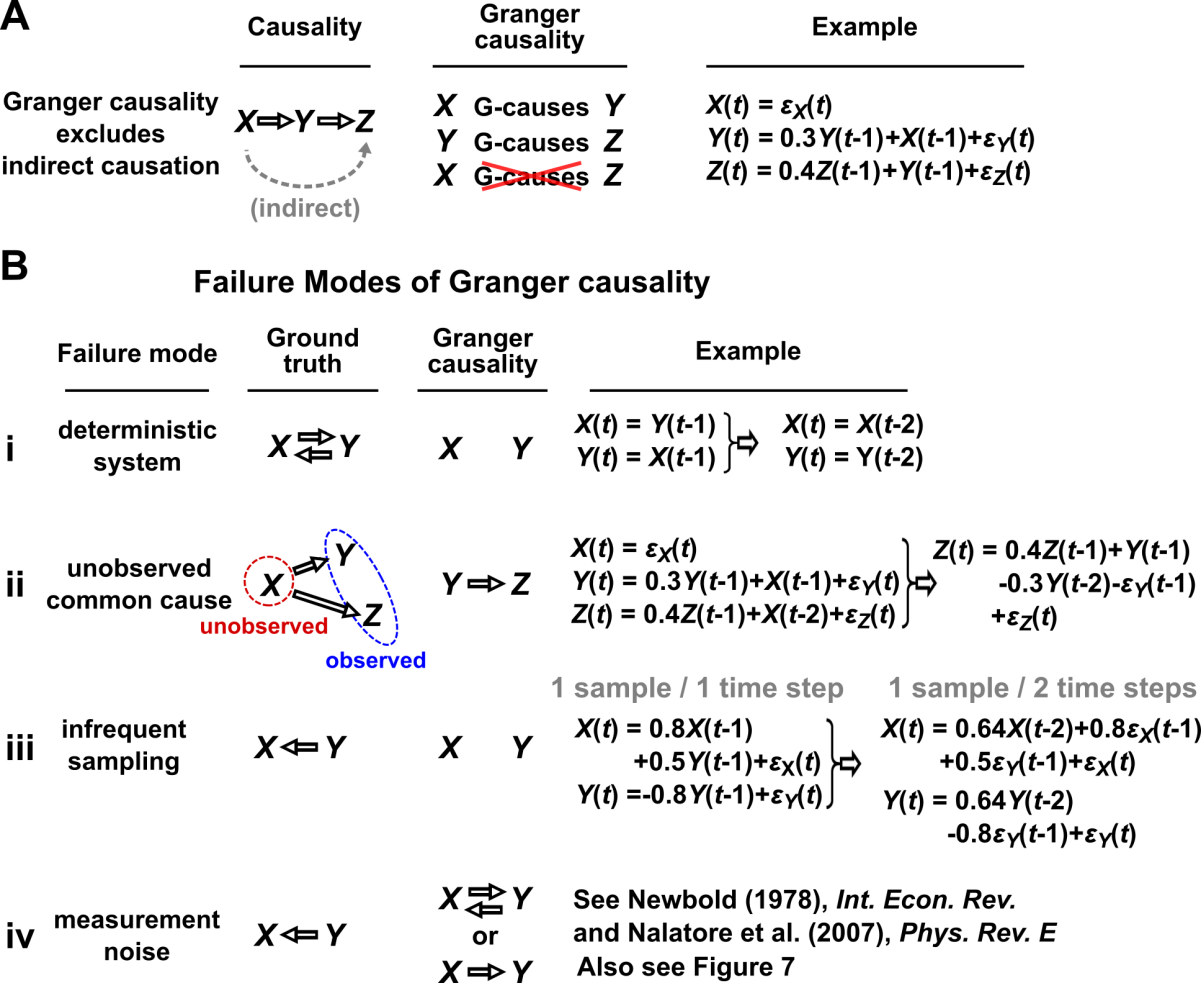
Causality versus Granger causality. (**A**) Granger causality is designed to reveal direct causes, not indirect causes. Although X causes Z, X does not Granger-cause Z because with the history of Y available, the history of X no longer adds value for predicting Z. This also shows that Granger causality is not transitive: X Granger-causes Y and Y Granger-causes Z, but X does not Granger-cause Z. (**B**) Failure modes of Granger causality when inferring direct causality. (**i**) False negative due to lack of stochasticity. X and Y mutually and deterministically cause one another through a copy operation (Ay and Polani, 2011; Peters et al., 2017): X⁢(t) copies Y⁢(t-1) and vice versa. Since X⁢(t-2) already contains sufficient information to know X⁢(t) exactly, the history of Y cannot improve prediction of X, and so Y does not Granger-cause X. By symmetry, X does not Granger-cause Y. (**ii**) False positive due to unobserved common cause. X causes Y with a delay of 1, and causes Z with a delay of 2. We only observe Y and Z. Since Y receives the same “information” before Z, the history of Y helps to predict Z, and thus Y Granger-causes Z, resulting in a false positive. (**iii**) Infrequent sampling can induce false negatives. Although there is a Granger causality signal when we sample once per time step, the signal is lost when we sample only once per two steps (Gong et al., 2015). (**iv**) Measurement noise can lead Granger causality to suffer both false positives and false negatives. $\epsilon_{X}(t)$, $\epsilon_{Y}(t)$, and $\epsilon_{Z}(t)$ represent process noise and are normal random variables with mean of 0 and variance of 1. All process noise terms are independent of one another.

In simple language, X is said to Granger-cause Y if a collection of time series containing all historical measurements predicts Y’s future behavior better than a similar collection that excludes the history of X. An important consequence of this definition is that Granger causality excludes indirect causes, as illustrated in Figure 3A. In practice, whether a causal relationship is direct or indirect depends on which variables are observed. For instance, in Figure 3A, if Y were not observed, then X would “directly” cause (and Granger-cause) Z.

Granger causality has many related but nonequivalent quantitative incarnations in the literature, including several that were proposed by Granger himself (Granger, 1969; Granger, 1980). Box 1 presents two definitions: one based on a linear regression which we call ‘linear Granger causality’ (Gibbons et al., 2017; Ai et al., 2019; Barraquand et al., 2020; Mainali et al., 2019) and another more general definition which we call ‘general Granger causality’ (also sometimes called nonlinear Granger causality; Granger, 1980; Diks and Panchenko, 2006; Bekiros and Diks, 2008; Vicente et al., 2011; Roux et al., 2013; Papana et al., 2017). See theorem 10.3 of Peters et al., 2017 for a discussion of the theoretical relationship between general Granger causality and (true) causality.

Box 1. **Granger causality**

**1. Linear Granger causality:**

Under linear Granger causality, X Granger-causes Y if including the history of X in a linear autoregressive model (Equation 1) allows for a better prediction of future Y than not including the history of X (i.e. setting all $\alpha_{k}$ coefficients to zero). By “linear autoregressive model”, we mean that the future value of variable Y is modeled as a linear combination of historical values of X and Y and all other observed variables that might help predict Y (“...”):

(1) $$Y_{t+1}=c+\sum_{k=0}^{n}\left(\alpha_{k}X_{t-k}+\beta_{k}Y_{t-k}+\cdots \right)+\varepsilon_{t}$$

Here, t is the time index, k=0,1,…,n is a time lag index, c is a constant, coefficients such as $\alpha_{k}$ and $\beta_{k}$ represent the strength of contributions from their respective terms, and $\varepsilon_{t}$ represents independent and identically-distributed (IID, Appendix 1) process noise (Figure 7A).

**2. General Granger causality (** Granger, 1980 **):**

Let $X_{t}$, $Y_{t}$, and $Z_{t}$ be series of random variables indexed by time t. X Granger-causes Y with respect to the information set $\left\{X_{t},Y_{t},Z_{t}\right\}$ if:

(2) $$P(Y_{t}|\{X_{k},Y_{k},Z_{k}\;for\;all\;k<t\})\neq P(Y_{t}|\{Y_{k},Z_{k}\;for\;all\;k<t\})$$

at one or more times t. Here, $P(Y_{t}|S)$ is the probability distribution of $Y_{t}$ conditional on the variable set S. Note that $Z_{k}$ in Equation 2 may include multiple variables and thus plays the same role as “. . .” in Equation 1.

### Granger causality failure modes

We discuss four important instances where Granger causality can fail as an indicator of direct causality (Figure 3B). These pathologies can be understood intuitively and can apply to both linear and general Granger causality. First, if a system has deterministic dynamics (see Appendix 3), then Granger causality may fail to detect causal relations (Figure 3Bi). More generally, if dynamics have a low degree of randomness, Granger causality signals can be very weak (e.g. knowing X’s past improves predictions of Y’s future only slightly; Janzing et al., 2013; Peters et al., 2017). Moreover, as we will discuss later, this limitation has motivated other methods that take a primarily deterministic view (Sugihara et al., 2012). Second, Granger causality may erroneously assign a direct causal relation between a pair of variables that have an unobserved common cause (Figure 3Bii). Third, recording data at a frequency below that of the original process by ‘subsampling’ (e.g. taking weekly measurements of a daily process) or by ‘temporal aggregation’ (e.g. taking weekly averages of a daily process) can alter the inferred causal structure (Figure 3Biii), although recent techniques can help with these issues (Gong et al., 2015; Hyttinen et al., 2016; Gong et al., 2017). Lastly, when measurements are noisy (Figure 3Biv), Granger causality can assign false interactions and also fail to detect true causality (Newbold, 1978), although some progress has been made on this front (Nalatore et al., 2007).

### Practical testing for linear and general Granger causality

One might still attempt to infer Granger causality despite the above caveats, especially in situations where caveats can be largely avoided. Linear Granger causality has standard parametric tests: if any of the $\alpha_{k}$ terms in Equation 1 is nonzero, then X linearly Granger-causes Y. Parametric tests are computationally inexpensive and available in multiple free and well-documented software packages (Seabold and Perktold, 2010; Barnett and Seth, 2014). These tests assume that time series are ‘covariance-stationary’, which means that certain statistical properties of the series are time-independent (Barnett and Seth, 2014; see also Appendix 3), and can fail when this assumption is violated (Toda and Phillips, 1993; Ohanian, 1988; He and Maekawa, 2001). Additionally, applying linear Granger causality to nonlinear systems can lead to incorrect causal conclusions (Li et al., 2018). One can assess whether the linear model (Equation 1) is a reasonable approximation, for instance by checking whether the model residuals $\varepsilon_{t}$ are uncorrelated across time (Feige and Pearce, 1979) as is assumed by Equation 1.

Tests for general Granger causality often use a statistic known as transfer entropy (Papana et al., 2012). Roughly, the transfer entropy from X to Y is the extent to which the entropy (a measurement of uncertainty) of Y’s future is reduced when we account for (specifically, condition on) the past of X (Schreiber, 2000; Cover and Thomas, 2006; Montalto et al., 2014; Papana et al., 2017). A significant transfer entropy thus indicates the presence of general Granger causality. Surrogate data are typically used to evaluate significance (Montalto et al., 2014; Papana et al., 2017; Shorten et al., 2021). However, the previously discussed surrogate data procedures are designed to test the null hypothesis of independence, which is different from the null hypothesis of general Granger non-causality (i.e. Equation 2, but replace ‘≠’ with ‘=’). More recent surrogate procedures have been proposed to address this issue (Runge, 2018a; Shorten et al., 2021). Several software implementations of Granger causality tests based on transfer entropy statistics are available (e.g. Montalto et al., 2014; Behrendt et al., 2019; Wollstadt et al., 2019).

Granger causality methods face challenges when datasets have a large number of variables (e.g. in microbial ecology). In this case, the summation in Equation 1 will contain a large number of terms, and so a regression procedure may fail to detect many true interactions (Runge et al., 2019a; Runge et al., 2019b). To handle systems with many variables, one can impose the assumption that only a small number of causal links exist (Gibbons et al., 2017; Mainali et al., 2019). This is sometimes called sparse regression or regularization. Additionally, under certain technical assumptions, it is possible to use a series of logical rules to remove unnecessary terms in a purely data-driven way (Runge et al., 2019b; Runge et al., 2019a). As an example, suppose that we wish to test whether pH is a Granger-cause of chlorophyll concentration in some aquatic environment and we infer based on a prior analysis that chlorophyll concentration is always independent of fluctuations in salinity. Then, most likely, salinity is irrelevant to the pH-chlorophyll relationship and can be safely omitted from our Granger causality analysis. As an aside, this reasoning could theoretically fail in pathological cases where, for instance, the ‘faithfulness’ condition (Appendix 2) is violated (see Example 7 of Runge, 2018b for a worked counterexample). These rules and their associated assumptions are formalized in ‘constraint-based’ causal discovery algorithms (Appendix 2; Peters et al., 2017; Glymour et al., 2019). The development of new causal discovery algorithms, and their application to time series, is a very active area of research (Hyvärinen et al., 2010; Runge et al., 2019b; Runge et al., 2019a; Sanchez-Romero et al., 2019).

## State space reconstruction (SSR): intuition, pitfalls, and implementations

The term ‘state space reconstruction’ (SSR) refers to a broad swath of techniques for prediction, inference, and estimation in time series analysis (Casdagli et al., 1991; Kugiumtzis et al., 1994; Asefa et al., 2005; Sugihara et al., 2012; Cummins et al., 2015). In this article, when we use the term SSR, we refer only to SSR methods for causality detection. The SSR approach is especially popular in empirical ecology (Brookshire and Weaver, 2015; Cramer et al., 2017; Hannisdal et al., 2017; Matsuzaki et al., 2018; Wang et al., 2019). SSR methods are intended to complement Granger causality: Whereas Granger causality has trouble with deterministic dynamics (Figure 3B), the SSR approach is explicitly designed for systems that are primarily deterministic (Sugihara et al., 2012). Since SSR is less intuitive than correlation or Granger causality, we introduce it with an example rather than a definition.

### Visualizing SSR causal discovery

**Figure 4.**
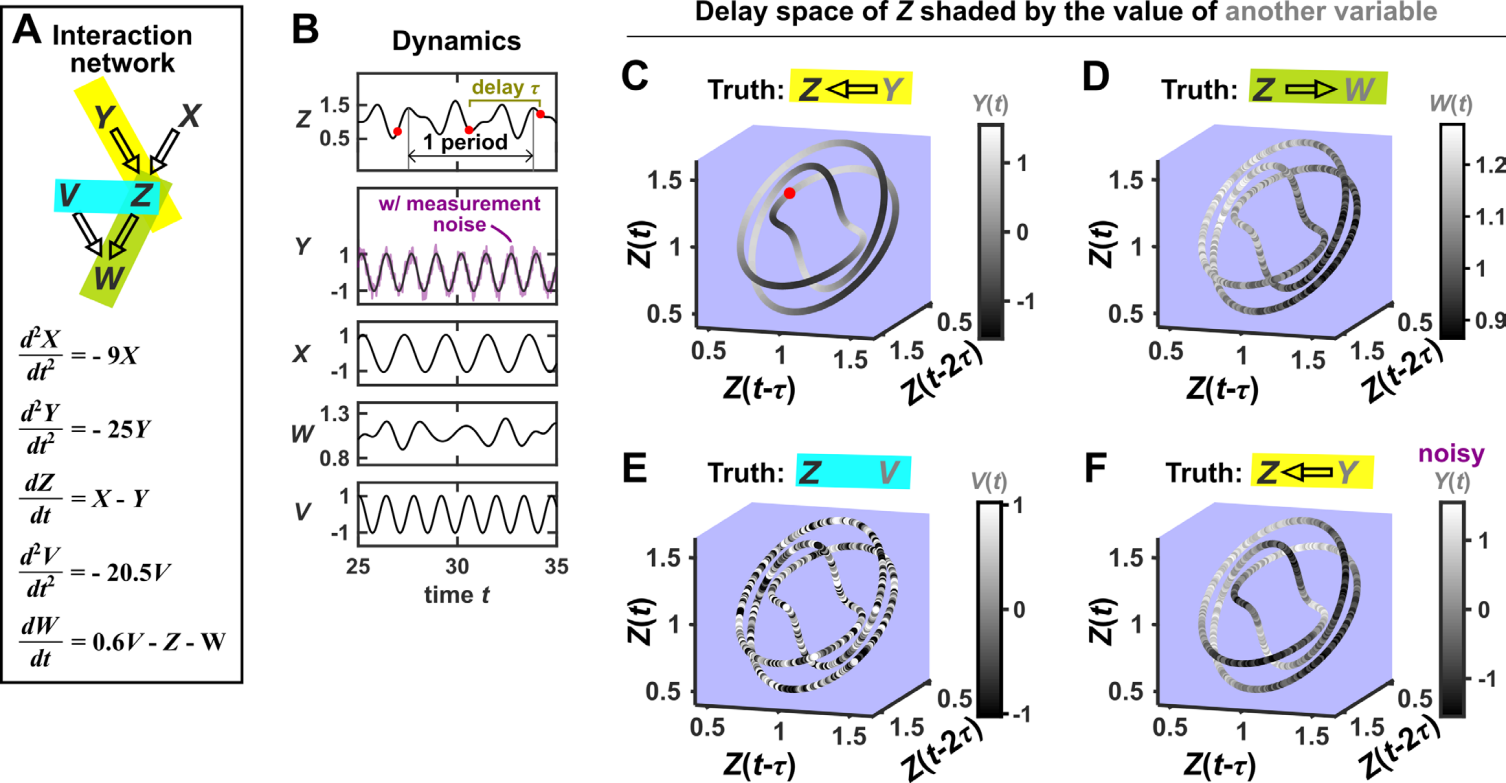
SSR causal methods look for a continuous map from the delay space of a causee to the causer, and this approach becomes more difficult in the presence of noise. (**A**) A toy 5-variable linear system. (**B**) Time series. The delay vector [Z⁢(t),Z⁢(t-τ),Z⁢(t-2⁢τ)] (shown as three red dots) can be represented as a single point in the 3-dimensional Z delay space (C, red dot). (**C**) We then shade each point of the Z delay space trajectory by its corresponding contemporaneous value of Y⁢(t) (without measurement noise). The shading is continuous (with gradual transitions in shade), which the SSR approach interprets as indicating that Y causes Z (correctly in this case). (**D**) When we repeat this procedure, but now shade the Z delay space trajectory by W⁢(t), the shading is bumpy, which the SSR approach correctly interprets to indicate that W does not cause Z. (**E**) Shading the delay space trajectory of Z by the causally unrelated V also gives a bumpy result. (**F**) Dynamics as in (**C**), but now with noisy measurements of Y (purple in B). The shading is no longer gradual. Thus with noisy data, inferring causal relationships becomes more difficult.

Consider the deterministic dynamical system in Figure 4. Here, Z is causally driven by X and Y, but not by W or V. We can make a vector out of the current value Z⁢(t) and two past values Z⁢(t-τ) and Z⁢(t-2⁢τ), where τ is the time delay and [Z⁢(t),Z⁢(t-τ),Z⁢(t-2⁢τ)] is called a ‘delay vector’ (Figure 4B, red dots). The delay vector can be represented as a single point in the three-dimensional Z ‘delay space’ (Figure 4C, red dot). We then shade each point of the trajectory in Z delay space according to the contemporaneous value of Y, which causally influences Z. Since in this example each point of the trajectory in Z delay space corresponds to one and only one Y⁢(t) value, we call this a ‘delay map’ from Z to Y. Notice that the Y⁢(t) gradient in this plot looks gradual in the sense that if two points are nearby in the delay space of Z, then their corresponding Y⁢(t) shades are also similar. This property is called ‘continuity’ (Appendix 4—figure 1). Overall, there is a continuous map from the Z delay space to Y, or more concisely, a ‘continuous delay map’ from Z to Y. A similar continuous delay map also exists from Z to its other causer X. On the other hand, if we shade the delay space of Z by W or V (neither of which causes Z), we do not get a continuous delay map (Figure 4D–E).

In this example, there is a continuous delay map from a causee to a causer, but not the other way around, and also no continuous delay map between causally unrelated variables. If this behavior reflects a broader principle, then perhaps continuous delay maps can be used to infer the presence and direction of causation. Is there in fact a broader principle?

In fact, there is a sort of broader principle, but it may not be fully satisfying for causality testing. The principle stems from a classic theorem due to Takens, 1980. A rough translation of Takens’ theorem is the following: If a particle follows a deterministic trajectory which forms a surface (e.g. an ant crawling all over a doughnut), and if we take one-dimensional measurements of that particle’s position over time (e.g. the distance from the ant’s starting position), then we are almost guaranteed to find a continuous delay map from our measurements (of current distance) to the original surface (the donut), as long as we use enough delays. (We walk through visual examples of these ideas in detail in Appendix 4.) A key result that follows from this theorem is that we can typically (‘generically’) expect to find continuous delay maps from ‘dynamically driven’ variables to ‘dynamically driving’ variables in a coupled deterministic dynamical system, as long as certain technical requirements are met (Cummins et al., 2015). Although the notion of ‘dynamic driving’ (Cummins et al., 2015) differs from our definition of causation, the two are related and we will still use the standard notion of causation when evaluating the performance of SSR methods. In theory, Takens’ theorem says that almost any choice of delay vector should work as long as it contains enough delays. However in practice, with finite noisy data, the behavior of SSR methods can depend on the delay vector selection procedure (Cobey and Baskerville, 2016; see also Appendix 4). Overall, Takens’ theorem and later results (Sauer et al., 1991; Cummins et al., 2015) form the theoretical basis of SSR techniques.

SSR techniques attempt to detect a continuous delay map (or a related feature) between two variables and use this to infer the presence and direction of causation (Sugihara et al., 2012; Ma et al., 2014; Harnack et al., 2017): A continuous delay map from Y to X is taken as an indication that X causes Y. The fact that the map points in the opposite direction as the expected causation is potentially counterintuitive. One informal explanation is that the delay vectors of the causee can contain a record of past influence from the causer (Sugihara et al., 2012). As a word of warning, while causation is one possible explanation for a continuous delay map, it is not the only possible explanation. Indeed, we now illustrate scenarios where a causal relationship and a continuous delay map do not coincide.

### SSR failure modes

**Figure 5.**
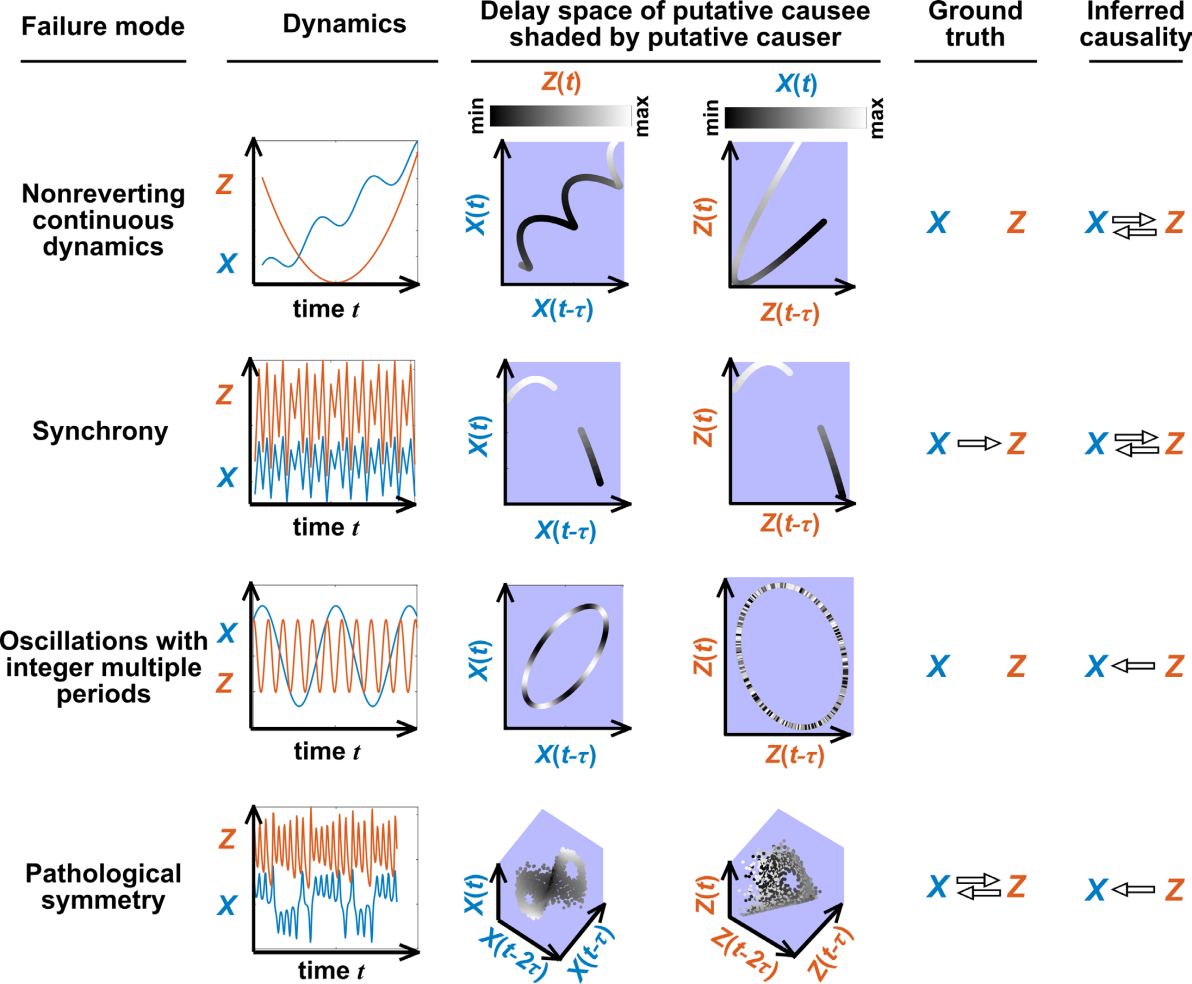
Failure modes associated with SSR-based causal discovery. Top row: Nonreverting continuous dynamics may lead SSR to infer causality where there is none. This example consists of two time series: a wavy linear increase and a parabolic trajectory. Although they are causally unrelated, we can find continuous delay maps between them. This is because there is (i) a continuous map from the delay vector [X⁢(t),X⁢(t-τ)] to t (X is ‘nonreverting’), and (ii) a continuous map from t to Z (Z is ‘continuous’), and thus there is a continuous delay map from X to Z (‘nonreverting continuous dynamics’; Appendix 4—figure 3). Thus, one falsely infers that Z causes X, and with similar reasoning that X causes Z. Second row: X drives Z such that their dynamics are ‘synchronized’, and consequently, we find a continuous delay map also from X to Z even though Z does not drive X. Note that the extent of synchronization is not always apparent from inspecting equations (e.g. Figure 12 of Mønster et al., 2017) or dynamics (row 5 of Appendix 4—figure 5). Third row: X oscillates at a period that is five times the oscillatory period of Z. There is a continuous delay map from X to Z even through X and Z are causally unrelated. Note that true causality sometimes also induces oscillations where the period of one variable is an integer multiple of the period of another (e.g. in Figure 4, the period of Z is three times the period of X). Bottom row: In the classic chaotic Lorenz attractor, X and Z cause one another, but we do not see a continuous map from the delay space of Z to X. This is because, as mentioned earlier, satisfying the conditions in Takens’ theorem makes a continuous mapping likely but not guaranteed (Appendix 4). Here, Z is an example of this lack of guarantee (Deyle and Sugihara, 2011) due to a symmetry in the system (see ‘Background definitions for causation in dynamic systems’ in the supplementary information of Sugihara et al., 2012).

**Figure 6.**
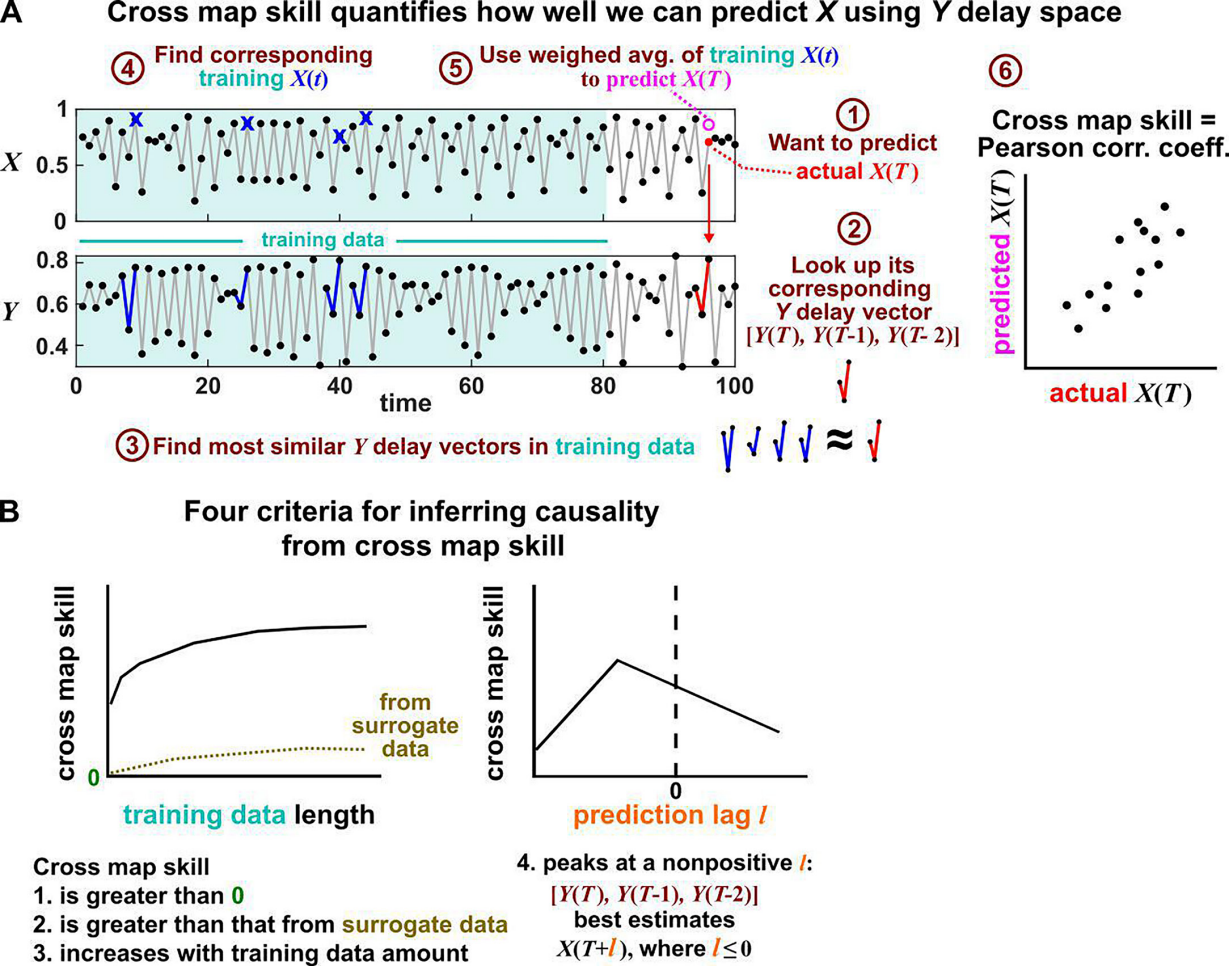
Illustration of the convergent cross mapping (CCM) procedure for testing whether X causes Y. (**A**) Computing cross map skill. Consider the point X⁢(T) denoted by the red dot (“actual X⁢(T)” in ①), which we want to predict from Y delay vectors. We first look up the contemporaneous Y delay vector [Y⁢(T),Y⁢(T-1),Y⁢(T-2)] (②, red dynamics), and identify times within our training data when delay vectors of Y were the most similar (i.e. least Euclidean distance) to our red delay vector (③, blue segments). We then look up their contemporaneous values of X (④, blue crosses), and use their weighted average to predict X⁢(T) (⑤, open magenta circle; weights are given as equations S2 and S3 in the supplement of Sugihara et al., 2012). We repeat this procedure for many choices of T and calculate the Pearson correlation coefficient between the actual X⁢(T) and predicted X⁢(T) (⑥). This correlation is called the “cross map skill”. While other measures of cross map skill, such as mean squared error, may also be used (Sugihara et al., 2012), here we follow the convention of Sugihara et al., 2012. (**B**) Four criteria for inferring causality from the cross map skill. Data points in (**A**) are marked by dots and connecting lines are visual aids.

Figure 5 illustrates four failure modes of SSR. In the first failure mode, which we refer to as ‘nonreverting continuous dynamics’ (top row of Figure 5; see also Appendix 4), a continuous map arises from the delay space of X to Z because a continuous map can be found from the delay space of X to time (‘nonreverting X’) and from time to Z (‘continuous Z’). This pathology leads to false causal conclusions and may explain apparently causal results in some early works where SSR methods were applied to data with a clear temporal trend. We are not aware of statistical tests for this problem, but Clark et al., 2015 recommend shading points in the delay space with their corresponding time to visually check for a time trend. In the second failure mode (Figure 5, second row; see also Sugihara et al., 2012), one variable drives another variable in such a way that the dynamics of the two variables are synchronized. Consequently, although the true causal relationship is unidirectional, bidirectional causality is inferred. Although the ‘prediction lag test’ (Figure 6B right panel) can sometimes alleviate this problem (Ye et al., 2015; Cobey and Baskerville, 2016), it is not foolproof as we demonstrate in Appendix 4. In the third failure mode (Figure 5 third row), X and Z both oscillate and X’s period is an integer multiple of Z’s period. In this case, Z is inferred to cause X even though they are causally unrelated (see also Cobey and Baskerville, 2016). In the fourth failure mode (Figure 5, bottom row), SSR gives a false negative error due to ‘pathological symmetry’, although this may be rare in practice.

### Convergent cross mapping: detecting SSR causal signals from real data

SSR causal discovery methods require testing for the existence of continuous delay maps between variables. However, testing for continuity in real data is complicated by noise and discrete sampling (Figure 4, compare panels C and F; see also Appendix 4—figure 1).

Several methods have been used to detect SSR causal signals by detecting approximate continuity (Cummins et al., 2015) or related properties (Sugihara et al., 2012; Ma et al., 2014; Harnack et al., 2017). The most popular is convergent cross mapping (CCM), which has been applied to nonlinear (Sugihara et al., 2012) or linear deterministic systems (Barraquand et al., 2020). CCM is based on a statistic called ‘cross map skill’ that quantifies how well a causer can be predicted from delay vectors of its causee (Figure 6A), conceptually similar to checking for gradual transitions when shading the causee delay space by causer values (Figure 4). Four criteria have been proposed to infer causality (Sugihara et al., 2012; Ye et al., 2015; Cobey and Baskerville, 2016; Figure 6B): First, the cross map skill must be positive. Second, the cross map skill must be significant according to some surrogate data test. Third, the cross map skill must increase with an increasing amount of training data. Lastly, the cross map skill must be greater when predicting past values of the causer than when predicting future values of the causer (the prediction lag test [Ye et al., 2015; Cobey and Baskerville, 2016] in the right panel of Figure 6B, but see Appendix 4 for caveats of this test). In practice, many if not most CCM analyses use only a subset of these four criteria (Sugihara et al., 2012; Brookshire and Weaver, 2015; Cramer et al., 2017; Wang et al., 2018). Other approaches to detect various aspects of continuous delay maps have also been proposed (Ma et al., 2014; Cummins et al., 2015; Harnack et al., 2017; Leng et al., 2020). We do not know of a systematic comparison of these alternatives.

## Simulation examples: external drivers and noise jointly influence causal discovery performance

In this section, we examine how environmental drivers, process noise, and measurement noise can influence the performance of Granger causality and CCM, using computer simulations. We constructed a toy ecological system with a known causal structure, obtained its dynamics (with noise) through simulations, and applied a linear Granger causality test (using the MVGC package of Barnett and Seth, 2014) and CCM (using the R language package rEDM) to test how well we could infer causal relationships.

**Figure 7.**
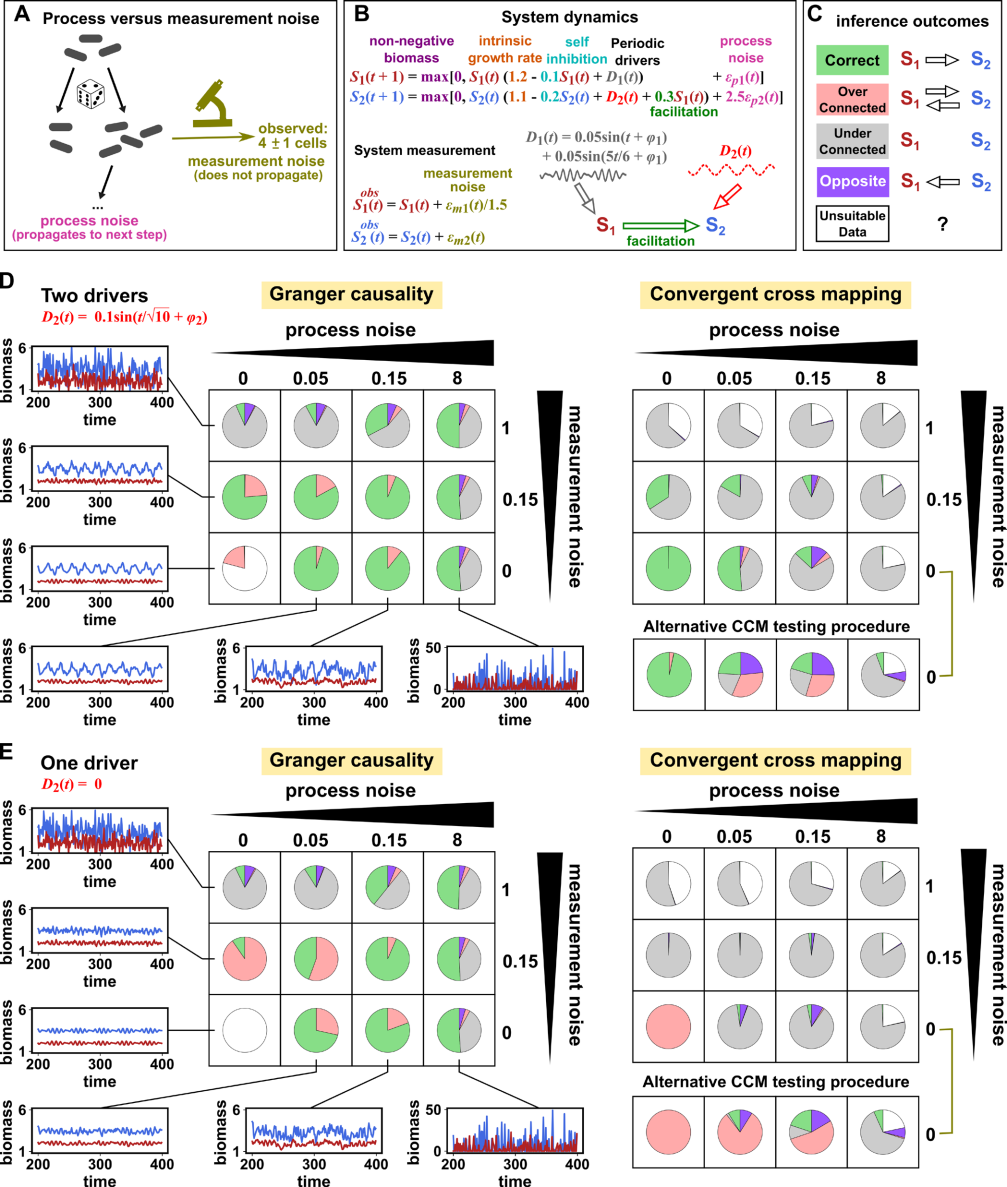
Performance of Granger causality and convergent cross mapping in a toy model with noise. (**A**) The effect of a time points’s process noise, but not its measurement noise, propagates to subsequent time points. (**B**) We simulated a two-species community. The process noise terms $\epsilon_{p\,1}\,\left(t\right)$ and $\epsilon_{p\,2}\,\left(t\right)$, as well as the measurement noise terms $\epsilon_{m\,1}\,\left(t\right)$ and $\epsilon_{m\,2}\,\left(t\right)$, are IID normal random variables with a mean of zero and a standard deviation whose value we vary. (**C**) Five possible outcomes of the causal analysis. (**D, E**) Community dynamics and causal analysis outcomes. We varied the level (i.e. standard deviation) of process noise and measurement noise. For Granger causality, we used the MVGC package (Appendix 5). For convergent cross mapping, we used the rEDM package to calculate cross map skill and to construct surrogate data, and custom codes for other tasks (Appendix 5). Each pie chart shows the distribution of inference outcomes from 1,000 independent replicates. Note that the MVGC package does not necessarily flag data corrupted by a problematic level of measurement noise (Lusch et al., 2016). In both the main and alternative CCM procedures, criterion 1 (positive ρ) was checked directly and random phase surrogate data were used to test criterion 2 (significance of ρ). Criterion 4 (prediction lag test) was not used, because the test is difficult to interpret for periodic dynamics where cross map skill can oscillate as a function of prediction lag length (Appendix 4—figure 5). The two procedures differ only in how they test criterion 3 (ρ increases with more training data): the main procedure uses bootstrap testing following Cobey and Baskerville, 2016 while the alternative procedure uses a Kendall’s τ as suggested by Chang et al., 2017.

We simulated a two-species community in which one species (*S*_1_) causally influences the other species (*S*_2_) but *S*_2_ has no influence on *S*_1_ (Figure 7B). Additionally, *S*_1_ is causally influenced by an unobserved periodic external driver and *S*_2_ either is (Figure 7D) or is not (Figure 7E) causally influenced by its own (also unobserved) periodic external driver. In an ecosystem, external drivers might appear as changes in temperature, light, or water levels, for example. We also added process noise to model the stochastic nature of natural ecosystems and added measurement noise to model measurement uncertainty. Process noise propagates to future times and can result from, for instance, stochastic migration and death (Figure 7A). In contrast, measurement noise does not propagate over time, and includes instrument noise as well as ecological processes that occur during sampling. Since tests for CCM causality criteria have varied widely (Cobey and Baskerville, 2016; Chang et al., 2017; Barraquand et al., 2020), we tested for CCM criteria using two different procedures (Figure 7 legend and Appendix 5).

Granger causality and CCM can perform well when their respective requirements are met, but both are fairly sensitive to the levels of process and measurement noise (Figure 7D and E, correct inferences colored as green in pie charts) and to details of the ecosystem (whether or not *S*_2_ has its own external driver; compare Figure 7D and E). In both methods, detection of the true causal link is disrupted by either the strongest measurement noise (standard deviation of 1) or the strongest process noise (standard deviation of 8) used here.

For Granger causality (Figure 7D and E, left panels), the MVGC package correctly rejects the data as inappropriate in the deterministic setting (lower left corner). When process and/or measurement noise is present, their relative amount is important: As measurement noise increases (from bottom to top), process noise often needs to increase (from left to right) for Granger causality to perform well. Indeed, prior analytical results (Newbold, 1978; Nalatore et al., 2007) show that measurement noise can induce false positives (e.g. red slices in row 2, column 2) and hide true positives (e.g. grey slices in row 1). Surprisingly, increasing measurement noise can sometimes improve performance (in column 3 of both panels, row two has a larger green slice than row 3).

To understand the CCM results (Figure 7D and E, right panels), recall that CCM is designed for deterministic systems, and fails when dynamics of variables are synchronized. When *S*_2_ has its own external driver (Figure 7D), there is no synchrony, and CCM performs admirably in the deterministic setting (lower left corner). CCM performs less well when measurement or process noise is introduced. Strikingly, when we remove the external driver of *S*_1_ (Figure 7E), CCM performs poorly. This is likely because the two species are now synchronized in the absence of noise (violating the ‘no synchrony’ requirement of CCM). However, adding noise, which removes the synchrony problem, violates the determinism requirement. So CCM is frustrated either way. Note that unlike CCM, Granger causality is less sensitive to the presence of underlying synchrony as long as this synchrony is disrupted by process noise. Additionally, the performance of CCM (Figure 7D and E, right panels) is sensitive to the test procedure (olive brackets).

In reality, where a system lies in the spectrum of process versus measurement noise is often unknown, and we are not aware of any method that reliably distinguishes between process noise and measurement noise without knowing the functional form of the system. Furthermore, how might one tell if a time series is stochastic or deterministic so that one can choose between Granger causality versus CCM? One idea is that deterministic processes tend to be more predictable than stochastic processes, at least in the short term (Hastings et al., 1993). Indeed, the inventors of CCM have recommended checking whether historical values of a time series can be used to accurately predict future values (Sugihara and May, 1990) before applying CCM (i.e. Clark et al., 2015). However, practical time series found in nature are most likely somewhere between the extremes of ‘fully deterministic’ (i.e. no measurement or process noise) and ‘fully stochastic’ (i.e. IID). Time series are often partly deterministic due to autocorrelation and partly stochastic due to random fluctuations. Indeed, simulations have found that SSR-based and Granger causality-based methods can both potentially succeed for such systems (Barraquand et al., 2020). Future work is needed to flesh out the nuances of when and why methods from these two classes provide similar or different performance (Barraquand et al., 2020).

## Summary: model-free causality tests are not assumption-free

We have described three causal discovery approaches for observational time series (Table 1). Although the techniques explored in this article have been called model-free and do not depend on prior mechanistic knowledge, they are by no means free from assumptions (Coenen et al., 2020). The danger that arises when we replace knowledge-based modeling with model-free inference is that we can replace explicitly stated assumptions with unstated and unscrutinized assumptions. Too frequently, both methodological and applied works fall into this trap. Nevertheless, when assumptions are clearly articulated and shown to be reasonable, model-free causal discovery techniques have the potential to jump-start the discovery process where little mechanistic information is known. Still, experimental follow-up (when possible) remains valuable since any technique that seeks to infer causality from observational measurements will typically require at least some assumptions that are difficult to fully verify.

**Table 1.** A comparison of three statistical causal discovery approaches.

	What does it mean if the method detects a link?	Implied causal statement	What are some possible failure modes?
Correlation	*X* and *Y* are statistically dependent.	*X* causes *Y*, *Y* causes *X*, or *Z* causes both.	Surrogate null model may make incorrect assumptions about the data-generating process.
Granger causality	The history of *X* contains unique information that is useful for predicting the future of *Y*.	*X* directly causes *Y*.	Hidden common cause; infrequent sampling; deterministic system (no process noise); excessive process noise; measurement noise
State space reconstruction	The delay space of *X* can be used to estimate *Y*.	*Y* causes *X.*	Nonreverting continuous dynamics; synchrony; integer multiple periods; pathological symmetry; measurement or process noise

We have discussed several failure modes of various causal discovery approaches (Table 1). Among these failure modes, measurement noise and nonstationarity have been repeatedly singled out as crucial considerations for real data (Stokes and Purdon, 2017; Barnett et al., 2018; Munch et al., 2020). While the deleterious effect of excessive measurement noise is intuitive, the pernicious effect of nonstationarity is not always appreciated. This is perhaps because the stationarity requirement, although ubiquitous, is sometimes hidden in the analysis pipeline. For example, when testing whether cross map skill (or correlation) is significant, surrogate data tests are commonly used (e.g. Lancaster et al., 2018), and nearly all of them require stationary data. Granger causality tests also typically require data to be stationary.

What comes next? We cannot cover all open fronts in data-driven causal discovery from time series, but do note a few directions that we think are important. First, given that practical ecological time series can rarely be shown to satisfy the assumptions of tests with mathematical exactness, we would benefit from a more complete understanding of how well tests for dependence and/or causality tolerate moderate deviations from assumptions. In a different direction, one may sometimes possess not a complete mathematical model, but instead some pieces of a model, such as the knowledge that nutrients influence the growth of organisms according to largely monotonic saturable functions. Techniques that attempt to make use of such partial models have recently obtained intriguing results (Daniels and Nemenman, 2015; Brunton et al., 2016; Mangan et al., 2016), and more would be welcome. Moreover, natural experiments often involve known external perturbations that are random or whose effects are poorly understood. An important question is how inference techniques might best take advantage of such perturbations (Eaton and Murphy, 2007; Rothenhäusler et al., 2015).

Perhaps most importantly, how can method developers best communicate their assumptions and caveats to method users who are potentially unfamiliar with technical terms or concepts? One effective strategy is to provide simulation examples of how applying techniques to pathological data may give incorrect results (Clark et al., 2015; Brunton et al., 2016). Video walkthroughs (e.g. Video 1; Brunton et al., 2017; Xie and Shou, 2021) may be another useful way to communicate how a method works as well as method assumptions. Finally, we recommend that editors and reviewers work with authors to ensure that failure modes and caveats are clearly articulated in the main text, along with accessible explanations of any necessary technical terms or concepts.

## Figures and Tables

**Appendix 1—figure 1.**
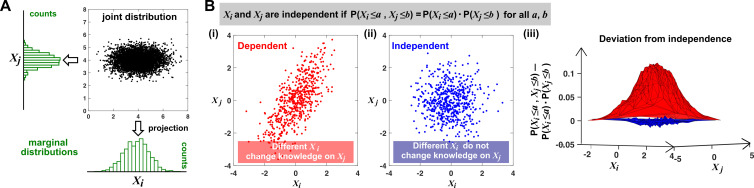
Joint distribution, marginal distributions, and dependence between two random variables. (**A**) A scatterplot of data associated with random variables $X_{i}$ and $X_{j}$ represents a ‘joint distribution’ (black). Histograms for data associated with $X_{i}$ and for data associated with $X_{j}$ represent ‘marginal distributions’ (green). Strictly speaking, joint and marginal distributions must be normalized so that probabilities (here represented as ‘counts’) sum to 1. Graphically, marginal distributions are projections of the joint distribution on the axes. Two random variables are identically distributed if their marginal distributions are identical. (**B**) Independence between two random variables. Gray box: a mathematical definition of independence, where ‘P‘ means probability. Two random variables are dependent if and only if they are not independent. Visually, if two random variables are independent, then different values of one random variable will not change our knowledge about another random variable. In (**i**), $X_{j}$ increases as increases (so that different values imply different expectations about $X_{j}$), and thus, $X_{i}$ and $X_{j}$ are not independent (i.e. they are dependent). In (**ii**), $X_{i}$ and $X_{j}$ are independent. One might argue that when $X_{i}$ values become extreme, $X_{j}$ values tend to land in the middle. However, this is a visual artifact caused by fewer data points at the more extreme $X_{i}$ values. If we had plotted histograms of $X_{j}$ at various $X_{i}$ values, we would see that $X_{j}$ is always normally distributed with the same mean and variance. (**iii**) Indeed, when we plotted the difference between the observed probability $P\left(X_{i}\leq a,X_{j}\leq b\right)$ and the probability expected from $X_{i}$ and $X_{j}$ being independent $P\left(X_{i}\leq a\right)\cdot P\left(X_{j}\leq b\right)$, (**ii**) showed a near-zero difference (blue), while (**i**) showed deviation from zero (red). This is consistent with $X_{i}$ and $X_{j}$ being independent in (**ii**) but not in (**i**).

**Appendix 1—figure 2.**
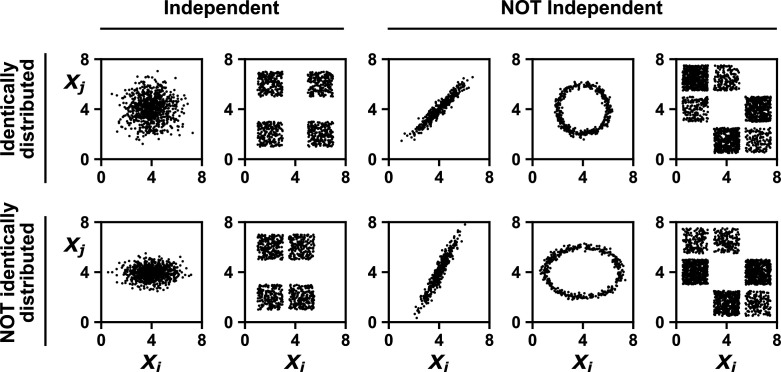
Examples of random variables that are identically distributed or not identically distributed, and independent or not independent. In the top row, $X_{i}$ and $X_{j}$ are identically distributed (projections of the scatter plot on both axes would have the same shape, as in Appendix 1—figure 1A). Note that in the top row of the rightmost column, the scatter plot is not symmetric along the diagonal line, yet projections on both axes yield identical marginal distributions: three segments of equal densities. Thus, the two random variables are identically distributed. In the bottom row, $X_{i}$ and $X_{j}$ are not identically distributed. In the leftmost two columns, the two random variables are independent (for more details about independence, see Appendix 1—figure 1B). In the last three columns, the two random variables are dependent: different $X_{i}$ values alter our knowledge of $X_{j}$.

**Appendix 1—figure 3.**
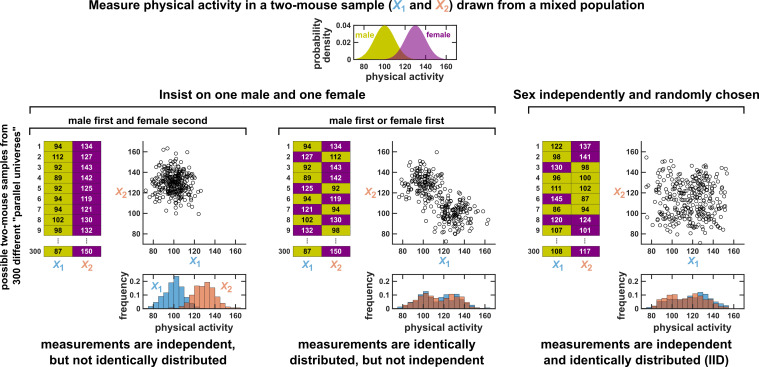
Measurements taken from a mixed population may still be IID, as long as sampling is independent and random. Consider a study in which physical activity is measured from a mixed population of low-activity male mice and high-activity female mice. For simplicity, suppose that the study uses only two mice. To see whether this could be an IID dataset, we imagine drawing many possible versions of that sample, and ask whether our first measurement $X_{1}$ and second measurement $X_{2}$ are identically distributed and independent. We could collect this sample in three different ways (3 sets of charts). On the left, we take our first measurement $X_{1}$ from a male and second measurement $X_{2}$ from a female. In this case, our two measurements are independent, but not identically distributed, and thus not IID. In the middle, we choose one male and one female per sample, but choose the first measurement randomly from a male or female. Now, our measurements are identically distributed but not independent (so also not IID). On the right, the sex of each measurement is randomly and independently chosen so that, for example, a sample might have two measurements from the same sex. In this case our sample is an IID dataset.

**Appendix 1—figure 4.**
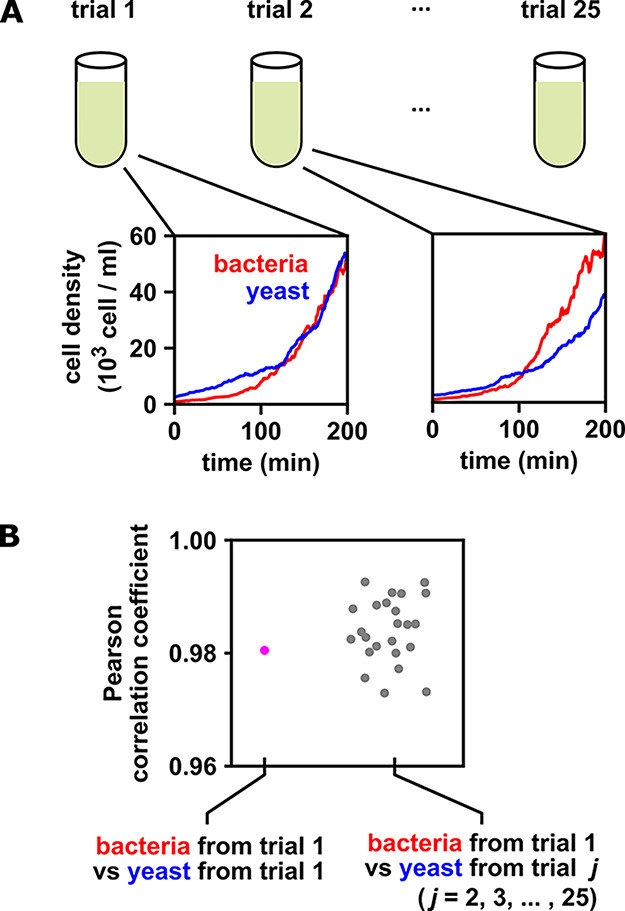
When multiple identical and independent trials are available, the significance of a correlation between time series within a trial can be assessed by comparing it to correlations between trials. (**A**) A thought experiment in which yeast and bacteria are grown in the same test tube, but follow independent dynamics. We imagine collecting growth curves from 25 independent replicate trials. (**B**) Correlations within and between trials. The Pearson correlation coefficient between yeast and bacteria growth curves from trial one is a seemingly impressive ∼0.98 (pink dot). But does this result really indicate that the two growth curves are dependent? To answer this question, notice that the yeast curves from other trials are similarly highly correlated to the bacteria curve from trial 1, even though they all come from independent trials (grey dots). Therefore, the pink dot cannot be used as evidence that the yeast and bacteria growth are dependent. If the within-trial correlation (pink dot) were stronger than, for instance, 95% of the between-trial correlations (grey dots), we would have evidence of dependence.

**Appendix 2—figure 1.**
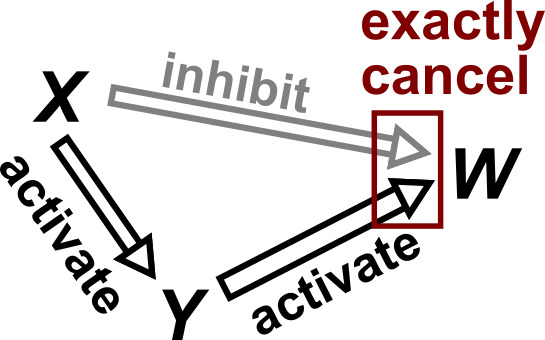
Violation of faithfulness condition due to precise cancellation of causal effects. Although X has a direct causal effect on W, we assume here that this is exactly canceled out by an opposing influence via the indirect path of X→Y→W. Thus, although the Markov condition does not require that X and W be independent, X and W are actually independent. We thus say that the joint probability distribution of the variables {X,Y,Z} is not faithful to the graph.

**Appendix 2—figure 2.**
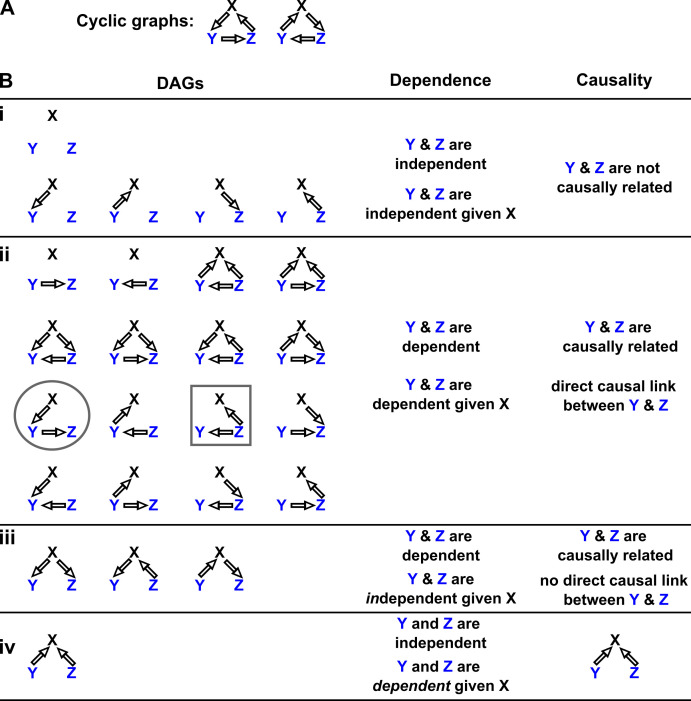
Probability distributions alone can specify causal structure to varying degrees of resolution. Consider a system of three and only three random variables X, Y and Z. Between each pair of variables, there are three possible unidirectional relationships: causation in one direction, causation in the opposite direction, and no causation. With three pairs of variables and three types of relationships, there are 3^3^ = 27 possible graphs. (**A**) Two of these graphs are cyclic, while the rest are DAGs. (**B**) If our system is described by a Markovian and causally faithful SEM, we can infer some aspects of causal structure from probability distributions alone. We demonstrate this by using the dependence relationships between Y and Z (blue) to infer causal relationships. (**Bi**): Y and Z are always independent. Y and Z are not causally related. (**Bii**): Y and Z are dependent, implying that they are causally related. (Recall that in this article, two variables are “causally related” if one causes the other, or they share a common cause.) Furthermore, Y and Z are conditionally dependent given X. For example, in the circled graph, variation in Y will affect Z, resulting in dependence between Y and Z, even if we control for X. (**Biii**): Y and Z are dependent, but are conditionally independent given X. There is no direct link between Y and Z, but they are causally related. Note that all three graphs are consistent with the following observations: Y and Z are dependent and conditionally independent given X; X and Z are dependent and conditionally dependent given Y; X and Y are dependent and conditionally dependent given Z. Thus, we cannot uniquely identify the causal structure from dependence relationships alone. (**Biv**): Y and Z are independent, but are conditionally dependent given X; see Appendix 2—figure 3 for an example of this scenario. This case corresponds to one and only one possible DAG.

**Appendix 2—figure 3.**
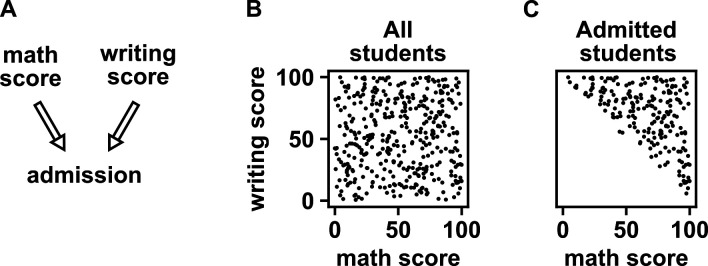
Selection bias creates the false impression of dependence. (**A**) DAG depicting the assumed causal relationship between math scores, writing scores, and admission to a certain college. (**B**) Math and writing scores in a fictitious student population are independent of each other, and take on random values distributed uniformly between 0 and 100. (**C**) A college admits a student if and only if their combined score exceeds 100. It is apparent that when we condition on college admission (by plotting only the scores of admitted students), math and writing scores show a negative association, indicating that they are dependent.

**Appendix 2—figure 4.**
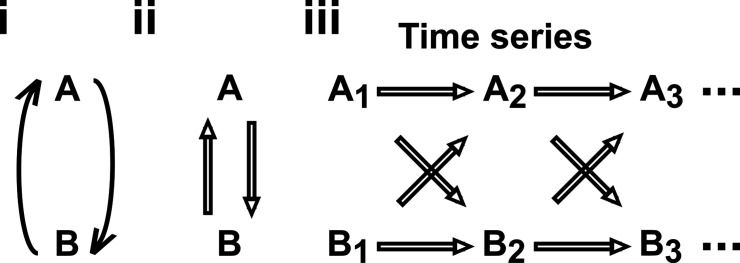
Causal discovery approaches designed for directed acyclic graphs (DAGs) can be applied to time series from systems with feedback. (**i**) Consider a mutualistic system where A and B represent the population sizes of two species that mutually facilitate each other’s growth. (ii) When the role of time is ignored, the causal graph is cyclic and thus not a DAG. (iii) For time series data where $A_{1},\;A_{2}$, … represent the population size of A at times 1,2,…, the causal graph is no longer cyclic since $A_{1}$ causes $B_{2}$ and $B_{1}$ causes $A_{2}$ and so on. Note that $A_{1}$ causes $A_{2}$ (and similarly $B_{1}$ causes $B_{2}$). This framework (Peters et al., 2017) has helped one of the authors classify mutations in ecological communities with feedback interactions (Hart et al., 2019; Hart et al., 2021).

**Appendix 3—figure 1.**
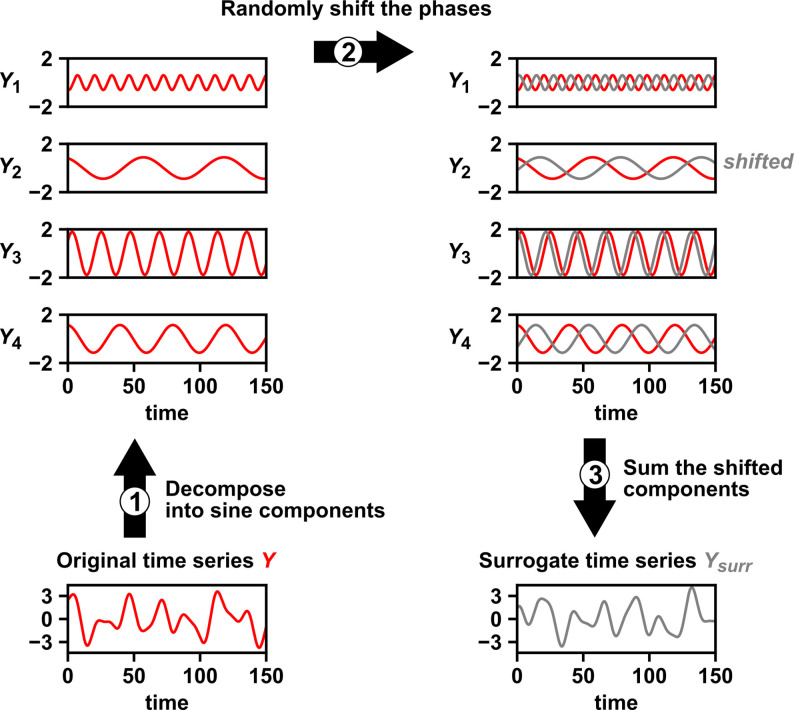
Intuition for random phase surrogate data methods. Random phase surrogate data methods generate $Y_{s\,u\,r\,r}$ by representing Y as a sum of sine waves (1), randomly shifting the phases of the component sine waves (2), and summing up the shifted sine waves (3).

**Appendix 3—figure 2.**
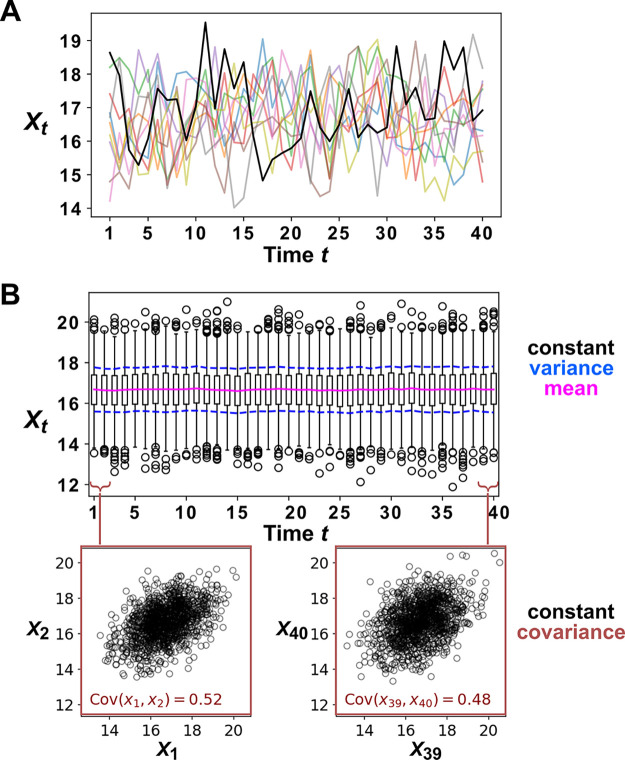
Example of a covariance-stationary process. (**A**) Ten replicate runs of the stochastic process described in Equation 4 with parameter choices a=0.6 and c=10. The noise term $\epsilon_{t}$ is a normal random variable with mean of zero and standard deviation of 1. To illustrate the behavior of a single replicate, we highlight one representative trajectory in black. (**B**) The distribution of $X_{t}$ values is shown for 1200 replicate runs of the same stochastic process as in (**A**). The mean of $X_{t}$ is given as a solid red line and the mean ± the standard deviation of $X_{t}$ is given by dashed blue lines. Bottom: $X_{t}$ is plotted against $X_{t+1}$ for two values of t.

**Appendix 3—figure 3.**
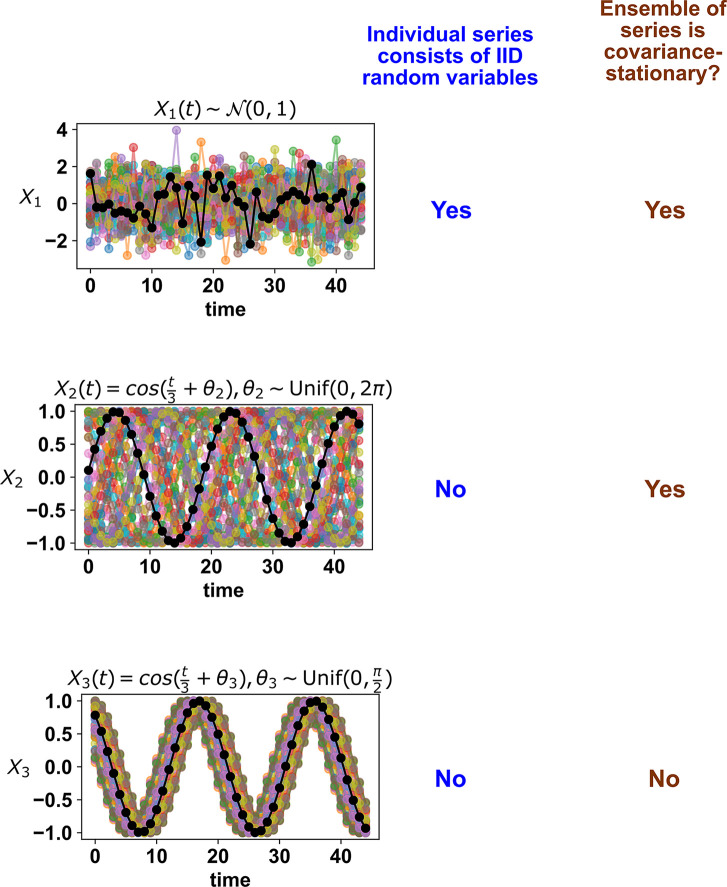
Whether a stochastic process is stationary depends on its entire ensemble of time series. The top panel shows IID standard normal noise. The middle and bottom panels both show sinusoidal curves. Although an individual time series from the middle panel looks similar to that from the bottom panel, only the middle panel shows a covariance-stationary process.

**Appendix 3—figure 4.**
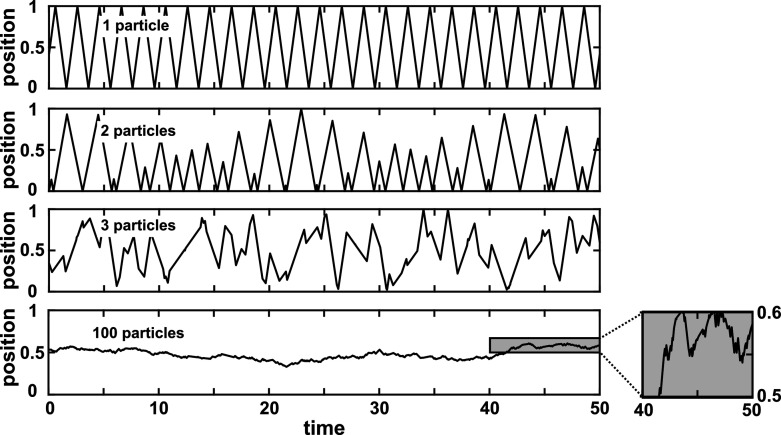
A many-variable deterministic system can be approximated as a stochastic system. The position of a particle in a system of particles bouncing in a one-dimensional box is plotted over time. In each simulation, particles with radius 0 bounce around in a box with walls of infinite mass placed at positions 0 and 1. Each particle has a mass of 1 and is initialized at a random position between 0 and 1 according to a uniform distribution. Initial velocities are chosen in the following way: The initial velocity of each particle in a box is first randomly chosen from between –1 and 1 according to a uniform distribution. Then, all initial velocities in a given box are multiplied by the same constant to ensure that the total kinetic energy of each box is 0.5. Kinetic energy is conserved throughout the simulation. The simulation then follows the particles as they experience momentum-conserving collisions with one another and with the walls.

**Appendix 4—figure 1.**
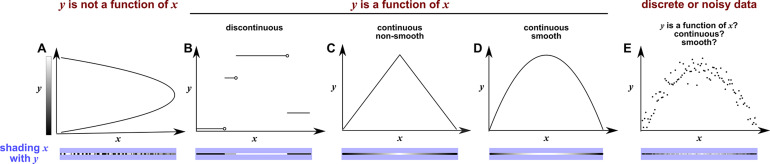
Continuity, smoothness, and the difficulty of evaluating the continuity or smoothness of a function with finite or noisy data. (**A**) y is not a function of x because a single x value can correspond to more than one y value. Here, when we shade x with the value of y, we randomly choose the upper or the lower y value, leading to bumpy shading. (**B**) y is a discontinuous function of x. This is because at any “breakpoint” (circle) between two adjacent segments, the limit taken from the left side is different from the limit taken from the right side. Shading x with y generates a ‘bumpy’ pattern. (**C**) y is a continuous function of x, and shading x with y generates a gradual pattern. (**D**) y is a continuous and smooth function of x. Consider any point on the curve (call it p). We can draw a line between p and a neighboring point to the left (*p*_*L*_). We can also draw a line between p and a neighbor to the right (*p*_*R*_). If the slopes of these two lines become equal as *p*_*L*_ and *p*_*R*_ become infinitesimally close to p, then the function is smooth. Although the function in (**C**) is continuous, it is not smooth since at the maximum point, the slope taken from the left-hand side is different from the slope taken from the right-hand side. A smooth function is always continuous. (**E**) With finite and noisy data, shading x with y often generates a bumpy pattern. It is unclear whether y is a function of x, and if yes, whether the function is continuous and/or smooth.

**Appendix 4—figure 2.**
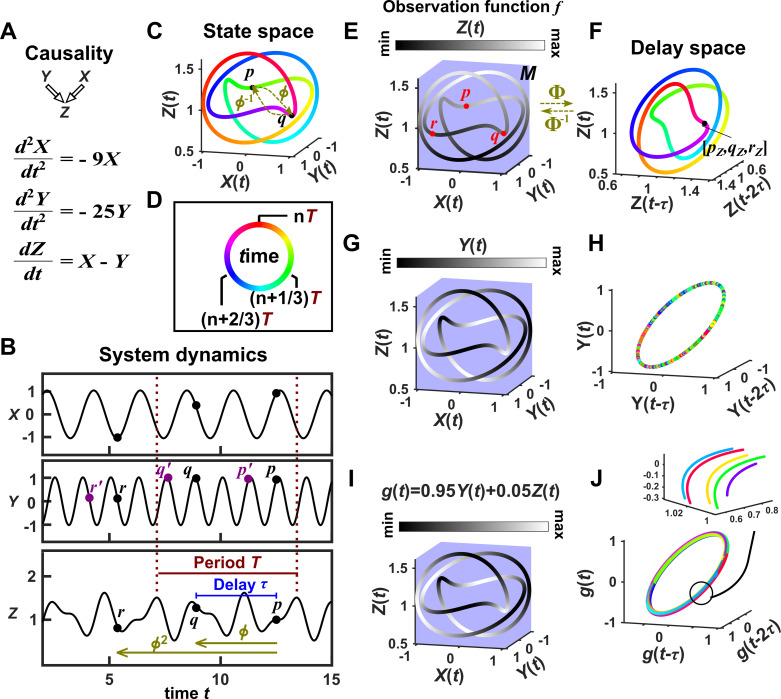
Illustration of Takens’ theorem. (**A**) We consider a 3-variable toy system in which X and Y causally influence Z, but Z does not influence X or Y. (**B**) Time series of the three variables. (**C**) We can represent the time series as the state space manifold M. Takens’ theorem requires that ϕ (a function that maps a point p at current time t to the point q at a previous time t-τ) and its inverse $\phi^{-1}$ (from past to current time) are both smooth ($C^{2}$: the first and second derivatives of the function exist and are continuous everywhere on the manifold). (**D**) To mark time progression, we color each point along the trajectory with its corresponding time value where time is represented as a color ring similar to a clock to reflect the periodic nature of system dynamics. (**E, G, I**) Shading the state space manifold with three different observation functions (f in Takens’ theorem) as indicated above each plot. (**F, H, J**) Delay space based on the observation function, colored with time. The map Φ in Takens’ theorem maps, for example, point p in panel E to point $\left[p_{Z},q_{Z,}\,r_{Z}\right]$ in panel F. The theorem tells us that for ‘generic’ choices of f and ϕ, the function Φ and its inverse $\Phi^{-1}$ provide a continuous mapping from the state space manifold to the delay space manifold, and vice versa. In this example τ=3.6. In panel J, multiple colors in a region are due to one period wrapping around the delay space multiple times (inset), but the color shading transition is continuous (similar to panel F).

**Appendix 4—figure 3.**
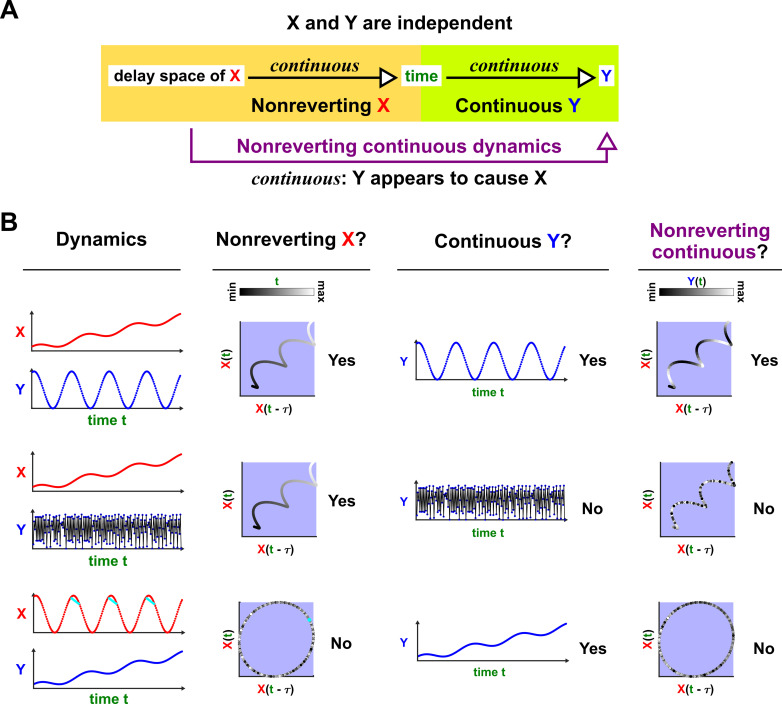
Nonreverting continuous dynamics. (**A**) Definition of nonreverting continuous dynamics. We call X nonreverting if the delay space of X maps continuously to t (time). We call Y ‘continuous‘ if Y⁢(t) is a continuous function of t. If X is nonreverting and Y is continuous then we say that the pair of time series (X,Y) has nonreverting continuous dynamics. (**B**) Examples. In each row, X and Y are causally independent. Leftmost column: Dynamics. Each red or blue dot (visible upon zooming in on some of the charts) represents a single time point. Second column: Looking for a continuous map from the delay vectors of X (the X delay space) to t, i.e. nonreverting X dynamics. Third column: Looking for a continuous map from t to Y by assessing whether Y at nearby times share similar values. Since the data occur at discrete times, the standard definition of continuity does not naturally apply, so ‘continuous Y’ really means ‘highly autocorrelated’. Fourth and final column: the presence or absence of ‘nonreverting continuous dynamics’. With nonreverting continuous dynamics, there is a continuous map from the X delay space to Y, and thus Y appears to cause X even though X and Y are causally independent.

**Appendix 4—figure 4.**
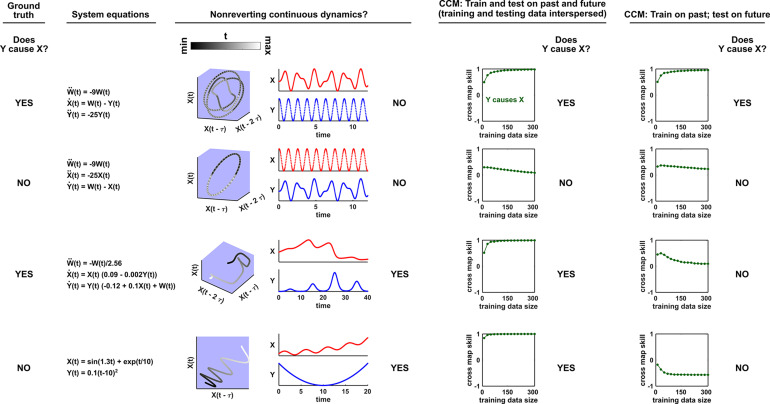
Nonreverting continuous dynamics impair the ability of CCM to correctly infer causality. Each row represents a system where Y does or does not causally influence X (Column 1). Column 2: Governing equations. Column 3: Checking for nonreverting continuous dynamics as in Appendix 4—figure 3. The top two rows do not have nonreverting continuous dynamics since there is no continuous map from the delay space of X to time. The bottom two rows have nonreverting continuous dynamics. Columns 4 and 5: Results of CCM where training and testing data are interspersed or when we train on the past and test on future. In the bottom two rows, CCM suffers false negative or false positive errors depending on the analysis details (e.g. whether training and testing data are interspersed).

**Appendix 4—figure 5.**
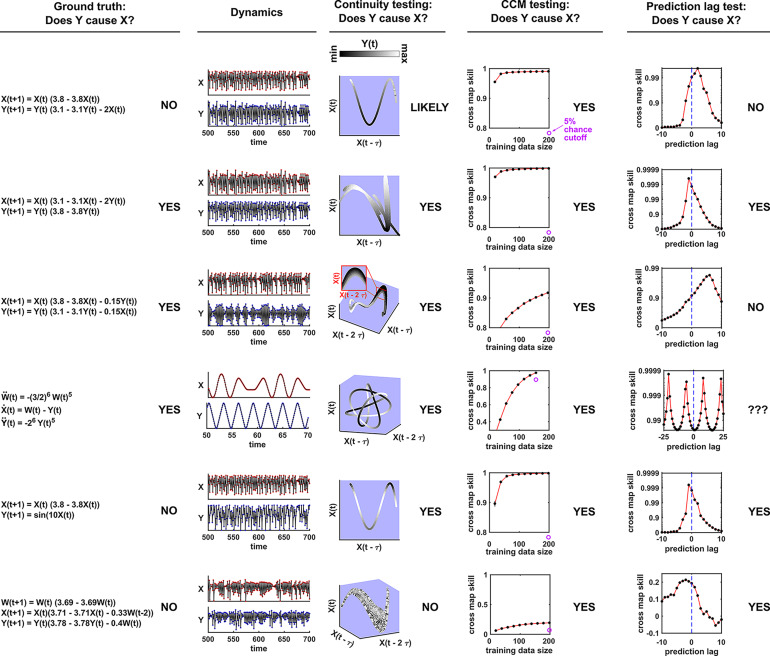
Comparison of visual continuity testing, cross map skill testing, and prediction lag testing in causal discovery. Each row represents a two-variable or three-variable system where Y does or does not causally influence X. The leftmost column shows the equations and ground truth causality. The second column shows a sample of X and Y dynamics. Red and blue dots represent X and Y values, respectively; black lines connecting the dots serve as a visual aid. The third column shows visual continuity testing and causal interpretation. We write ‘likely’ in the top row because the map from X delay space to Y appears to have some small bumps on the right side of the plot. The fourth column shows cross map skill testing (without the prediction lag test) and causal interpretation. Black dots show cross map skill. Open purple dots show the 5% chance cutoff at the maximum library size according to random phase surrogate data testing (see Appendix 5), or are placed below the horizontal axis if the 5% chance cutoff is below the plot. In all systems Y appears to cause X according to cross map skill testing since cross map skill is positive, increases with training data size, and is significant according to the surrogate data test. The rightmost column shows the prediction lag test and causal interpretation.

**Appendix 4—figure 6.**
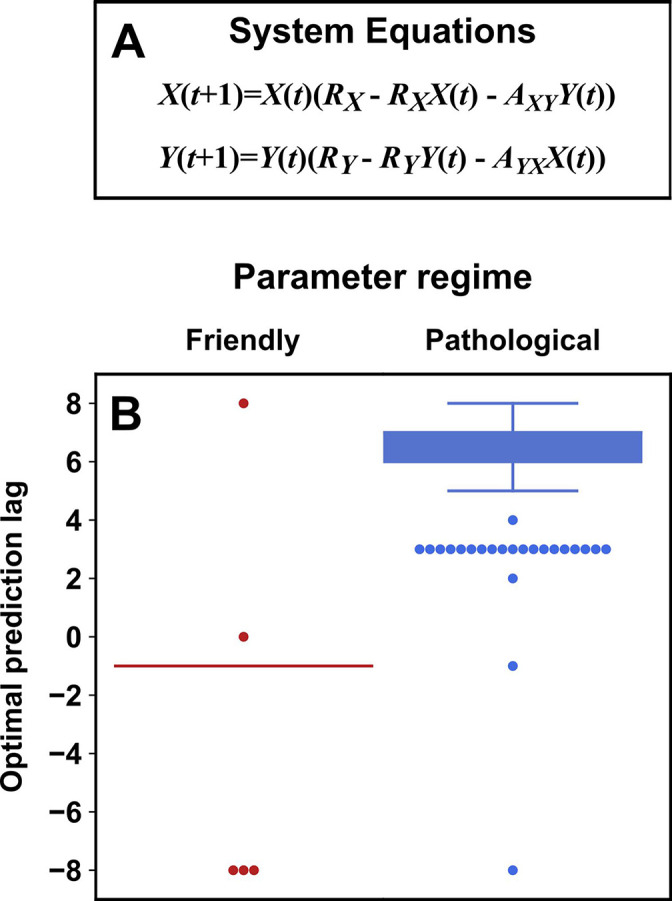
Parameters within a ‘pathological’ regime almost always cause the prediction lag test to erroneously reject a true causal link. (**A**) System equations. For both ‘friendly’ and ‘pathological’ regimes, initial conditions X⁢(1) and Y⁢(1) were independently and randomly drawn from the uniform distribution between 0.01 and 0.99 (“U⁢n⁢i⁢f⁢(0.01,0.99)”), and *R*_*X*_ was drawn from U⁢n⁢i⁢f⁢(3.7,3.9). *R*_*Y*_ was drawn from U⁢n⁢i⁢f⁢(3.7,3.9) (‘friendly’) or U⁢n⁢i⁢f⁢(3.1,3.3) (‘pathological’). $A_{X\,Y}$ and $A_{Y\,X}$ were independently drawn from U⁢n⁢i⁢f⁢(0.05,0.1) (‘friendly’) or U⁢n⁢i⁢f⁢(0.15,0.2) (‘pathological’). (**B**) Box plots show the optimal prediction lag when using delay vectors made from X to predict values of Y in 250 systems with parameters selected randomly as described just now. In the ground truth model for this system, Y exerts a causal influence on X. In the ‘friendly’ parameter regime, the optimal prediction horizon is negative, correctly indicating that Y does indeed cause X. In the ‘pathological’ regime, the optimal prediction horizon is positive, and so the prediction lag test would wrongly conclude that Y does not cause X. In the friendly regime the ‘box’ is shown as a line because the vast majority of trials had the same optimal prediction lag of -1.

## Additional files

Source code 1. Code for simulations.

## Appendix 1

### Random variables and their relationships

#### Dependence between random variables and between vectors of random variables

The concepts of dependence and independence between random variables are central to many statistical methods, including those that concern causality. A random variable is a variable whose values or experimental measurements depend on outcomes of a random phenomenon and follow a particular probability distribution. Reichenbach’s common cause principle states that if X and Y are random variables with a statistical dependence (such as a nonzero covariance), then one or more of three statements is true: X causes Y, Y causes X, or a third variable Z causes both X and Y. The common cause principle cannot be proven from the axioms of probability; rather, the principle is itself a fundamental assumption that supports much of the modern statistical theory of causality (Section 1.4.2 of Pearl, 2000).

As an example, consider the size and length of a bacterial cell. If a larger cell tends to be longer, then cell volume and cell length covary and are thus dependent. A mathematical definition of dependence (and its opposite, independence) is presented in Appendix 1—figure 1B.

Dependence can be readily generalized from the definition in Appendix 1—figure 1 to become a property between two vectors of random variables. (Note that a time series can be viewed as a vector of random variables.) For example, suppose that we measure two variables X and Y over two days. Our (very short) time series are then $\left[X_{1},X_{2}\right]$ and $\left[Y_{1},Y_{2}\right]$ where the subscript index denotes the day of measurement. Similar to Appendix 1—figure 1B, we would say that our two time series are independent if

$$P(X_{1}\leq x_{1},\;X_{2}\leq x_{2},\;Y_{1}\leq y_{1},\;Y_{2}\leq y_{2})=P(X_{1}\leq x_{1},\;X_{2}\leq x_{2})\;P(Y_{1}\leq y_{1},\;Y_{2}\leq y_{2})$$

for all choices of $x_{1},x_{2},y_{1},y_{2}$.

#### When are two random variables independent and identically distributed (IID)?

Many statistical techniques require repeated measurements that can be modeled as independent and identically distributed (IID) random variables, and passing non-IID data (such as time series) into such techniques can lead to spurious results (e.g. Figure 2; see also Koplenig and Müller-Spitzer, 2016). Random variables are IID if they have the same probability distribution and are independent (Appendix 1—figure 1). In Appendix 1—figure 2 we give examples of pairs of random variables that are (or are not) identically distributed, and that are (or are not) independent. Note that two dependent random variables can be linearly correlated (Appendix 1—figure 2, 3rd column), or not (Appendix 1—figure 2, 4th column).

Random sampling from a population with replacement is one way to produce “IID data” (which we use as a shorthand for “data which can be modeled as IID random variables”). For example, repeatedly rolling a standard die can be thought of as randomly sampling from the set {1,2,3,4,5,6} with replacement: if the first trial registers 1, then the second trial can register one as well. Otherwise, if sampling was done *without* replacement, then the second trial must not register 1, which means that the outcome of the second trial would depend on the outcome of the first trial.

#### A sample drawn from a mixed population can still be IID, as long as sample members are chosen randomly and independently

Since the IID concept is so central to statistical analysis, we wish to further clarify one conceptual difficulty that may arise. To set the stage, suppose that a scientist measures the levels of voluntary physical activity in a collection of mice that includes both males and females. Also suppose that female mice tend to be more physically active than male mice (Rosenfeld, 2017). Since this dataset now contains measurements from both the less active males and the more active females, we might naively think that these data cannot be IID.

In fact, such a dataset still might be IID, but this depends on how the scientist chooses which mice to measure. To illustrate this fact, consider the highly simplified scenario in which only two mice are assayed for physical activity. Let $X_{1}$ and $X_{2}$ be random variables that describe the activity levels of these two mice. We consider three different ways that the scientist might select which mice to assay. Only one of these ways will result in an IID dataset.

First, suppose that the scientist chooses to measure *X*_1_ from a male mouse and *X*_2_ from a female mouse. In this case, to see whether *X*_1_ and *X*_2_ are IID, we can use the same visualization strategy as in Appendix 1—figure 1. That is, we imagine many possible ‘parallel universes’, each with a different possible two-mouse dataset (left panel of Appendix 1—figure 3). This allows us to visualize the joint distribution of *X*_1_ and *X*_2_. We can then see that *X*_1_ and *X*_2_ are independent, but not identically distributed.

Second, suppose that the scientist again selects exactly one mouse of each sex, but randomizes the order so that both *X*_1_ and *X*_2_ have an equal chance of being measured from a male or female mouse (middle panel of Appendix 1—figure 3). We can now see that *X*_1_ and *X*_2_ are identically distributed, but not independent.

Lastly, suppose that the scientist selects mice randomly, and without any information about whether a mouse is male or female. In this case, the two-mouse sample might be all male, all female, or have one of each. Once again we plot the joint distribution of *X*_1_ and *X*_2_ by imagining their values across many different parallel universes (right panel of Appendix 1—figure 3). We then see that that *X*_1_ and *X*_2_ are finally independent identically distributed. Overall, a set of measurements can be IID even if they are taken from a mixed population, as long as they are sampled randomly from among different subpopulations.

#### Independence and statistical conditioning

Here, we first restate the concept of independence in terms of statistical conditioning, and then introduce the related concept of conditional independence.

It is intuitive that two variables are independent if knowledge of one variable tells us nothing about the other. The statistical notion of independence captures this intuition: Random variables X and Y are independent if the conditional distribution of X given Y is always equal to the marginal distribution of X. For discrete random variables, this condition can be written

(3) P(X=x|Y=y)=P(X=x)

or equivalently written P(X=x,Y=y)=P(X=x)P(Y=y) for all x and y. For continuous random variables, independence can be written in terms of probability density functions as $f_{X}\,\left(x|y\right)=f_{X}\,\left(x\right)$ or equivalently, $f_{X,Y}\,\left(x,y\right)=f_{X}\,\left(x\right)\,f_{Y}\,\left(y\right)$ where $f_{X}\,\left(x|y\right)$ is the conditional density of X given Y, $f_{X,Y}\,\left(x,y\right)$ is the joint density of X and Y, and $f_{X}\,\left(x\right)$ and $f_{Y}\,\left(y\right)$ are the marginal densities of X and Y, respectively.

The statement “X and Y are conditionally independent given Z” intuitively means that X and Y are independent when we only analyze outcomes where Z has a certain value. For discrete random variables, this condition is written P(X=x|Y=y,Z=z)=P(X=x|Z=z), or equivalently, P(X=x,Y=y|Z=z)=P(X=x|Z=z)P(Y=y|Z=z), for all x, y, and z. For continuous random variables, we have a similar formulation except that the probability P is replaced by the probability density f (i.e. $f_{X,Y}\,\left(x,y|z\right)=f_{X}\,\left(x|z\right)\,f_{Y}\,\left(y|z\right)$ for all x,y,z). If X and Y are not conditionally independent given Z, then X and Y are conditionally dependent given Z.

One could be forgiven for worrying about the feasibility of testing for dependence between long time series. This is because as a time series grows longer, the amount of data needed to get a sense of its probability distribution would seem to grow extremely rapidly. Thus, when X and Y are vectors that represent long time series, estimating the distributions in Equation 3 seems unrealistic. However, establishing that two time series are dependent only requires that we show that the distributions on the left and right sides of Equation 3 differ. Showing that two distributions differ can be much easier than actually estimating those distributions. For instance, if we know that the averages of two univariate distributions are different, then we immediately know that the two distributions are not the same, even if we know nothing about their shapes. Indeed, Appendix 1—figure 4 demonstrates a way to test for dependence between time series with only a moderate number of replicates, and without any assumptions about the shapes of the distributions. Additionally, surrogate data methods can be used to test for dependence with only one replicate of each time series, as discussed in the main text.

## Appendix 2

### Causal discovery with directed acyclic graphs

#### Discovering causal relationships and their associated directed acyclic graphs (DAGs)

Many theoretical results and data-driven methods for causal analysis begin by representing causal relationships as a directed acyclic graph (DAG). That is, one makes a graph by represeting random variables as nodes and by drawing a directed edge from each direct cause (or parent) to its causee (or child), as in Figure 1B; additionally, the graph is acyclic, meaning that that it does not contain any directed paths from any variable back to itself. The acyclicity condition is often required for nice theoretical properties and ease of analysis (Spirtes and Zhang, 2016). Additionally, when data are temporal, a particular node in the graph commonly refers to a particular variable measured at a particular time (e.g. chapter 10 of Peters et al., 2017). If we follow this convention and note that causation cannot flow backward in time, and if we additionally exclude instantaneous causation, then our causal graph will be acyclic, even for systems with feedback (Appendix 2—figure 4).

DAGs are useful visual tools in their own right, but for many purposes we need to be more mathematically precise about what we mean when we draw an edge from one variable to another. Thus, often one interprets a causal DAG as corresponding to a set of equations with the following two conditions: First, each variable can be written as a function of (only) the variable’s direct causers and a random process noise term unique to the variable. Models that satisfy this condition are called structural equation models (SEMs) (Hitchcock, 2020b). Second, all process noise terms are (jointly) independent of one another. SEMs that satisfy this second condition are called Markovian and have a useful property called the ‘causal Markov condition’ (Pearl, 2000). (Some authors [Peters et al., 2017], but not all [Hitchcock, 2020b], require that all SEMs be Markovian by definition.) The causal Markov condition, along with the related ‘causal faithfulness condition’ are key assumptions that allow one to connect statistical structure to causal structure and infer aspects of causal structure from data, even in observational settings.

The causal Markov condition states that if there is no path from X to Y in a DAG (i.e. we cannot go from X to Y by following a sequence of edges in the forward direction), then X and Y are conditionally independent given X's parents (Pearl, 2000; Zhang and Spirtes, 2008). In this context Y can be either a variable or a set of variables. As an example, consider the boxed DAG in Appendix 2—figure 2B. Here, X and Y share the common cause Z. Each variable depends on its parents, and on its own process noise term. Although X and Y are dependent, the causal Markov condition expresses the intuitive idea that if we were to control for Z, then X and Y would become independent. Note that if X does not have any parents, then the statement “X and Y are conditionally independent given X's parents” reduces to “X and Y are independent”.

The causal faithfulness condition is, like the Markov condition, very useful in causal discovery and often quite reasonable. However, faithfulness is more difficult to state precisely and concisely without first introducing technical notation such as ‘d-separation’ (as in definition 6.33 of Peters et al., 2017). We attempt to give the gist of the idea here and direct readers to other sources (Peters et al., 2017; Zhang and Spirtes, 2008) for more precise definitions. The causal faithfulness condition is a kind of converse to the causal Markov condition. Recall that the causal Markov condition requires certain conditional (or unconditional) independence relationships based on the causal graph structure. Let us call any other independence relationships (i.e. those not required directly or indirectly by the causal Markov condition) ‘extra’ independence relationships. The (joint) probability distribution of random variables is causally faithful to the DAG if no ‘extra’ independence relationships exist (Hitchcock, 2020b). An imprecise shorthand for the faithfulness condition is ‘independence relationships indicate the absence of certain causal relationships’. The faithfulness condition can be violated when two effects precisely cancel each other (Appendix 2—figure 1).

Existing observational causal discovery methods for the IID (e.g. non-temporal) setting are diverse. Such methods can differ greatly in the assumptions they make (e.g. whether there are hidden variables or ‘unknown shift interventions’), the reasoning they employ, and the resolution of causal detail they provide (e.g. a unique causal graph versus a set of several plausible graphs) (Heinze-Deml et al., 2018). We will briefly introduce two classes of causal methods: (1) constraint-based search and (2) structural equation models (SEMs) with assumptions about the functional forms of equations (Spirtes and Zhang, 2016). However, these two classes, while illustrative of different modes of causal discovery, are far from an exhaustive list (Heinze-Deml et al., 2018).

Constraint-based search uses independence and dependence relationships (and their conditional counterparts) to narrow down the scope of possible causal graphs without exhaustively checking all possibilities (which can be enormous in number even for a handful of variables). The PC algorithm (named after its inventors Peter Spirtes and Clark Glymour) and the fast causal inference algorithm are examples of constraint-based search methods (Glymour et al., 2019). However, constraint-based methods often find multiple graphs that are consistent with the same set of data (e.g. Appendix 2—figure 2Biii, see legend; see also Spirtes and Zhang, 2016).

Functional form-based (or SEM-based) approaches to causal discovery begin by assuming a particular functional form for causal relationships, and then assess a given causal hypothesis by inspecting the joint distribution between a potential causer and its potential causee (Spirtes and Zhang, 2016). These methods rely on the fact that in a Markovian SEM, each variable has a noise term that is independent of the noise terms of all other variables (Peters et al., 2017). Given two dependent variables with no hidden common causes, one can use an appropriate regression to estimate values of a proposed causee based on the proposed causer (Spirtes and Zhang, 2016). If the residuals of this regression are independent from the proposed causer, then the proposed causal direction is consistent with the data (Hoyer et al., 2008). Crucially, theoretical results indicate that for a fairly wide variety of scenarios (e.g. linear non-Gaussian and post-nonlinear models), we can expect the data to be consistent with only one causal direction, thus enabling unambiguous identification of the causal direction (Spirtes and Zhang, 2016). An illustrative graphic example is given in Figure 3 of Spirtes and Zhang, 2016 and also in Figure 3 of Glymour et al., 2019. Similar ideas can be applied to multivariate systems (Hoyer et al., 2008; Peters et al., 2012).

## Appendix 3

### Mathematical concepts for stochastic time series

#### Stationarity

Many methods for time series analysis require that data satisfy some stationarity condition, meaning that certain statistical properties of the data must remain constant across time. Two important stationarity conditions are strong stationarity and covariance-stationarity. A stochastic process $X_{t}$ is called strongly stationary if the joint distribution of any k consecutive values $\left(X_{t},X_{t+1},\cdots ,X_{t+k-1}\right)$ is independent of time t(Definition 20.1 of Greene, 2012). A stochastic process $X_{t}$ is covariance-stationary (or weakly stationary) if: (1) the ensemble mean $E[X_{t}]$ is finite and does not depend on t; (2) the variance $\mathrm{Var}\,\left[X_{t}\right]$ is finite and does not depend on t; (3) for all choices of h, the covariance $\mathrm{Cov}\,\left(X_{t},X_{t+h}\right)$ is finite and does not depend on t (Definition 20.2 of Greene, 2012).

As an illustrated example of a covariance-stationary process, consider a population whose dynamics are governed by death and stochastic migration (similar to Figure 2C):

(4) $$X_{t}=\left(1-a\right)\,X_{t-1}+c+\epsilon_{t}$$

Here, *X*_*t*_ is the population size at time t, a is the proportion of the population lost due to death during one time step, c is the average number of individuals migrating into the population during one time step, and $\epsilon_{t}$ is a random variable with a mean of zero which represents temporal fluctuations in the number of migrants. Suppose that we observed the dynamics of 10 populations governed by Equation 4 such that the populations all have the same parameters, but are independent (Appendix 3—figure 2A). Then, at each time point t, we will have some distribution of values of *X*_*t*_. In fact, if we have not just 10, but 1,200 replicates, we can see that the distribution of values of *X*_*t*_ does not appear to depend on time (Appendix 3—figure 2B, top). Furthermore, the covariance between *X*_*t*_ and $X_{t+1}$ does not appear to depend on time either (Appendix 3—figure 2B, bottom).

Although it is common to talk about a time series being stationary or nonstationary, this is technically a slight abuse of language. Just as the mean and variance are properties of a random variable (and not of any single data point obtained from that random variable), stationarity is a property of a stochastic process (and not of any single time series produced by that process). This fact is illustrated by comparing the middle and bottom rows of Appendix 3—figure 3. If we examine any one time series from the middle or bottom rows (e.g. the black curves in each), we see that they have essentially the same dynamics (i.e. they are sine waves with the same frequency). However, the process shown in the middle row is covariance-stationary (as shown below), whereas the process shown in the bottom row is not since its mean changes over time.

To see that the middle row of Appendix 3—figure 3 shows a covariance-stationary process, we can show that the mean, variance, and covariance of the process are independent of time:

$$E[X_{2}(t)]=\frac{1}{2\pi }\int_{0}^{2\pi }cos\left(\frac{t}{3}+\theta_{2}\right)d\theta_{2}=0$$

$$Var[X_{2}(t)]=\frac{1}{2\pi }\int_{0}^{2\pi }{\left(cos\left(\frac{t}{3}+\theta_{2}\right)\right)}^{2}d\theta_{2}=\frac{1}{2}$$

$$Cov(X_{2}(t),X_{2}(t+h))=\frac{1}{2\pi }\int_{0}^{2\pi }cos\left(\frac{t}{3}+\theta_{2}\right)cos\left(\frac{t+h}{3}+\theta_{2}\right)d\theta_{2}=\frac{1}{2}cos\left(\frac{h}{3}\right)$$

#### Deterministic processes with many variables may appear stochastic

A deterministic time series from a system with many variables can be approximated as stochastic. This is illustrated below in Appendix 3—figure 4. When we track the trajectory of a particle in a box with 99 other particles (Appendix 3—figure 4 bottom row), the observed trajectory appears random, even though the governing equations of motion are deterministic. In particular, the motion of our particle over each time step can be approximated as having a random component. Note that this flavor of randomness is in general different from the phenomenon called chaos. In chaotic dynamics, each time step needs not be random, but small changes in initial conditions lead to large changes at later times.

## Appendix 4

### State space reconstruction

#### Difficulty of evaluating the continuity or smoothness of a function with finite or noisy data

##### Considerations for selecting delay vector parameters for SSR

To construct delay vectors for SSR, one must choose the delay vector length E and the time delay τ. How does one choose E and τ? In general, detecting a continuous delay map requires that the delay vector length E be large enough so that no two parts of the delay space cross. For example, using E=2 (instead of E=3) to make Figure 4C would have projected the delay space onto two dimensions. This would introduce line crossings, which would in turn produce artifactual discontinuities in the shading. On the other hand, the amount of data required to perform SSR inference is said to grow with the delay vector length (Sugihara et al., 2012). SSR is less sensitive to τ, although it is possible to mask a continuous delay map by choosing a “bad” τ. For example, consider what would happen to Figure 4C if we set τ to the period of Z. Since the delay vector is [Z⁢(t),Z⁢(t-τ),Z⁢(t-2⁢τ)], setting set τ to the period of Z would force all 3 elements of the delay vector to always be equal. In geometric terms, this would compress the delay space onto a line, destroying the continuous delay map. However, bad choices of τ such as this are rare. Various practical methods are available for systematically choosing E and τ, and delay vectors with variable delays (e.g. [Z⁢(t),Z⁢(t-2),Z⁢(t-7)]) have also been used (Harnack et al., 2017; Cobey and Baskerville, 2016; Jia et al., 2020).

##### Historical notes on the basis of SSR

Takens’ celebrated paper (Takens, 1980) was a major theoretical advance that has inspired a variety of data-driven methods for both causality detection and forecasting (e.g. Perretti et al., 2013). Theorem 1 of Takens, 1980 is reproduced below. Here, the term ‘map’ can be used interchangeably with ‘function’: a map from X to Y sends each point in X to exactly one point in Y.

Appendix 4—box 1. Takens’ theorem. Takens’ theorem (theorem 1 of Takens, 1980): Let M be a compact manifold of dimension m. For pairs (ϕ,f),ϕ:M→M a smooth diffeomorphism and f:M→R a smooth function, it is a generic property that the map $\Phi_{\left(\phi ,f\right)}:M\to R^{2m+1},$ defined by

$$\Phi_{\left(\phi ,f\right)}(p)=f(p),f(\phi (p)),...,f(\phi^{2m}(p))$$

is an embedding; by “smooth” we mean at least $C^{2}$. Authors’ note: A function is in the class “$C^{k}$” if its *k*th derivative is continuous.

We will attempt to illustrate Takens’ theorem using the example in Appendix 4—figure 2. This example consists of a deterministic dynamical system with three variables X, Y, and Z . To begin, we visualize the state space in XYZ coordinates (Appendix 4—figure 2C), and color the trajectory with time (a colored clock-like ring in Appendix 4—figure 2D to highlight the periodic nature of system dynamics). This trajectory is the manifold M in Takens’ theorem and is 1-dimensional (m=1) since it is a loop. Takens’ theorem then asks us to choose a function ϕ, which we will define as a function that “points into the past”. Specifically, ϕ is a function that maps a point p on the manifold M at the current time t to the point q at a previous time t-τ. Similarly, $\phi^{2}\,\left(p\right)$ would apply ϕ twice and map p at the current time t to the point r at time t-2⁢τ (olive in Appendix 4—figure 2B), and $\phi^{-1}\,\left(q\right)$ would map point q at the past time t-τ to the point p at the current time t (Appendix 4—figure 2C). Note that ϕ and $\phi^{-1}$, which are ‘discrete-time’ mappings, are distinct from the differential equations that generated the system dynamics (which are continuous in time; Appendix 4—figure 2A). The term ‘diffeomorphism’ in the theorem means that both this function ϕ and its inverse function (the map from past to present) are smooth (Appendix 4—figure 1).

The next symbol in the theorem is f, which can be viewed as an ‘observation’ function that maps each point on the manifold to a single real number (e.g. in Appendix 4—figure 2E, $f\,\left(p\right)=p_{Z}$ so that f simply returns the Z coordinate of point p). Takens’ theorem then asks us to consider a function Φ that maps a point p at time t on our state space manifold (Appendix 4—figure 2E) to a point in the ‘delay space’. The coordinates of the delay space are given by applying the observation function to point p (which occurs at at time t), point q (at time t-τ), and point r (at time t-2⁢τ), so that a single point in the delay space is $\left[p_{Z},q_{Z},r_{Z}\right]$ with respect to a particular time t (Appendix 4—figure 2F). This choice of delay space comes from three earlier choices: First, we consider delayed values of Z since Z is what our observation function f returns; second, since m (the dimension of the manifold) is 1, the delay space should be of dimension 3 (=2⁢m+1) per Takens’ theorem; third, the delay length of τ comes from our diffeomorphism ϕ. Then, Takens’ theorem states that for ‘most’ (technically, ‘generic’) choices of f and ϕ, the function Φ is an embedding. This means that Φ is diffeomorphic to its image. That is, the curve in delay space will map smoothly (and thus continuously) to the manifold M and vice versa (Huke, 2006).

Indeed from Appendix 4—figure 2C-F, we can see that for our choice of observation function (i.e. f=Z⁢(t)), there is a map from the state space manifold M to the delay space manifold. This is because each dot in the state space manifold corresponds to a single time color (i.e. a point within a period), and each time color corresponds to a single dot in the delay space manifold, and thus, each point in the state space manifold corresponds to a single point in the delay space manifold. Moreover, Φ is continuous because the maps from state space to the time ring and from the time ring to delay space are both continuous. Similarly, we can see that the inverse of Φ, which points from the delay space manifold to the state space manifold is also a continuous map, as guaranteed (generically) by Takens’ theorem.

Strikingly, if the observation function is Y, we will no longer have a continuous map from the delay space trajectory (now of Y) to the state space trajectory. This is visualized as ‘bumpy coloring’ in Appendix 4—figure 2H. In fact, we cannot even map the delay space to the time ring or the state space: (p,q,r) and $\left(p^{\prime },q^{\prime },r^{\prime }\right)$ occupy the same point in the Y delay space, yet correspond to different times within a period (Appendix 4—figure 2B) and thus they correspond to different locations in the state space. Takens’ theorem took care of this pathology using the word ‘generic’. That is, Y is not considered a generic observation function here. On the other hand, if we use an observation function based on 95% Y mixed with 5% Z, we get an embedding from the state space to the delay space (Appendix 4—figure 2I–J). This is essentially what the term ‘generic’ means in the context of topology: Although some observation functions do not give you an embedding, these ‘bad’ observation functions can be tweaked just a tiny bit to become ‘good’ ones. Similarly, some choices of ϕ do not work (i.e. τ=T for this system), but these are exceptions (see Theorem 2 of Huke, 2006 for what makes a ϕ “generic”).

At a conceptual level, SSR causality inference can be performed by shading the delay space of one variable (the potential causee) with the contemporaneous value of another variable (the potential causer), and inferring a causal link if this shading is continuous. In the example of Figure 4 in the main text, shading the delay space of Z with Y generates a continuous pattern, consistent with Y causing Z. On the other hand, shading the delay space of Z with W shows a bumpy pattern, consistent with W not causing Z.

Sauer and colleagues (Sauer et al., 1991) later extended Takens’ theorem by proving a similar result that is in some ways more general. Theorem 2.5 in Sauer et al., 1991 is distinct but related to Takens’ theorem, and applies to cases that Takens’ theorem does not cover, such as fractal spaces. Additionally, (Sauer et al., 1991) replaces the concept of ‘generic’ functions with a different notion (‘prevalence’), which is closer to a probabilistic statement. Cummins et al., 2015 then formally connected these results to a notion of potentially causal coupling between dynamic variables.

##### Nonreverting continuous dynamics: criteria and effects on convergent cross mapping

We first illustrate ‘nonreverting continuous dynamics’, which reflects a nonstationarity pathology for SSR techniques. We then discuss how nonreverting continuous dynamics affects CCM.

We use the phrase ‘nonreverting continuous dynamics’ to describe the following idea: If the X delay space maps continuously to t (‘nonreverting’ X), and t maps continuously to Y⁢(t) (‘continuous’ Y), then the X delay space will map continuously to Y⁢(t), even if X and Y are causally unrelated (Appendix 4—figure 3A). Appendix 4—figure 3B illustrates this with three scenarios in which X and Y are causally *independent* time series. In the top row, the X delay space maps continuously to t and t maps continuously to Y⁢(t) , so we get nonreverting continuous dynamics and a continuous delay map from X to Y even though X and Y are independent. In the middle row, the X delay space maps continuously to t, but t does not map continuously to Y⁢(t), so we do not have nonreverting continuous dynamics (i.e. no continuous map from the X delay space to Y). In the bottom row, the X delay space does not map continuously to t. This is because a single delay vector (shown as a cyan dot in the delay space) occurs at multiple times (shown as repeated cyan line segments whose starting and ending points denote the two values of the delay vector), generating a bumpy pattern similar to Appendix 4—figure 1A. In this case, even though t maps continuously to Y⁢(t), we do not have nonreverting continuous dynamics and we do not get a spurious continuous map from the X delay space to Y.

Nonreverting continuous dynamics interferes with CCM causal discovery. Although one could attempt to mitigate the nonstationarity problem by interspersing training and testing data before quantifying cross map skill (Luo et al., 2015; Appendix 4—figure 4, Column 4), we find that this approach leads to false positive errors (Appendix 4—figure 4, bottom row). In contrast, the alternative (not interspersing training and testing data) can lead to false negative errors (Appendix 4—figure 4, third row). Thus, the ability to correctly infer causality with CCM is vastly reduced when data exhibit nonreverting continuous dynamics.

##### The prediction lag test: intuition and some failure modes

State space reconstruction methods suffer false positive errors in the presence of synchrony (Sugihara et al., 2012). This occurs when “the dependence of the dynamics of the forced variable on its own state is no longer significant” (Sugihara et al., 2012). Ye et al. proposed a test in an effort to solve this problem (Ye et al., 2015). Their procedure relies on finding mappings from the delay vector [X(t),X(t−τ),X(t−2τ),...,X(t−(E−1)τ)] to Y⁢(t+l), where E is the delay vector length, τ is the time lag, and l is a key variable known as the “prediction lag”. They then examine how the cross map skill (Figure 6B) varies with the prediction lag. According to this technique, if the cross map skill is maximized at a positive prediction lag (l>0), then the putative causality is spurious and arose from, for example, strong unidirectional forcing. The reasoning is that if the causee were to predict the future of the causer, then causation would appear to flow backward in time, which is nonsensical. On the other hand, if the highest quality mapping occurs at a non-positive prediction lag (l≤0), then we have further evidence that the detected causality is real and not spurious (Ye et al., 2015).

We find that while this test correctly distinguishes between real and spurious causal signals at some times, at other times it does not. Within each row of Appendix 4—figure 5, we examine a different system and ask whether Y causes X according to: (1) the ground truth model, (2) our visual continuity test, (3) a CCM cross map skill test (without the prediction lag test), and (4) the prediction lag test.

In rows 1 and 2 of Appendix 4—figure 5, the prediction lag test performs well, overturning the results of the visual continuity and CCM tests when apparent causality is spurious (row 1), and agreeing with the continuity and CCM tests (row 2) when apparent causality is real (modified from Ye et al., 2015 Equation 2). However in row 3, the prediction lag test dismisses a true causal link as spurious. Moreover, when we apply the prediction lag test to a system with a periodic putative driver (row 4), we find that cross map skill is a periodic function of the prediction lag. While this result is what we would expect mathematically, its causal interpretation is unclear. The fifth row of Appendix 4—figure 5 is an extreme case of strong forcing, where Y⁢(t+1) is a function of X⁢(t), but not Y⁢(t). Here the prediction lag test gives a false positive error. In the bottom row, X and Y do not interact, but are both driven by a common cause W with different lags. Specifically, W⁢(t) exerts a direct effect on Y⁢(t+1) and on X⁢(t+3). Thus, Y receives the same information as X, but at an earlier time, analogous to Figure 3ii. Consistent with this, delay vectors of X predict past values of Y better than future values of Y. Thus, the prediction lag test produces a false positive error.

Ye et al., 2015 applied the prediction lag test to 500 systems with the same form as in the third row of Appendix 4—figure 5 but with randomly chosen parameters. They found that within the parameter range they sampled, false negative errors as in Appendix 4—figure 5 do occur, but such errors are rare. We repeated the randomized numerical experiment from Ye et al., 2015 for both the original parameter range of Ye et al., 2015; Appendix 4—figure 6B, ‘friendly’ parameter regime and a second parameter range of the same volume in parameter space (Appendix 4—figure 6B, ‘pathological’ parameter regime). In this pathological parameter regime, false negative errors occur in the overwhelming majority of cases.

## Appendix 5

### Detailed methods

#### Methodological details for Figure 2

For panel B, we simulated the random walk system

X⁢(t+1)=X⁢(t)+ϵ⁢(t)

where the ϵ⁢(t) terms were drawn independently from a normal distribution with mean of 0 and standard deviation of 1. We simulated this system from the initial condition of X⁢(1)=0 through 999 subsequent steps. For panel C, we simulated the autoregressive system

X⁢(t+1)=0.75⁢X⁢(t)+10+ϵ⁢(t)

where the ϵ⁢(t) terms were again drawn independently from a normal distribution with mean of 0 and standard deviation of 1. We simulated this system from the initial condition of X⁢(1)=40 for 1999 subsequent steps. We only used the final 1000 steps for computing the correlation between two time series.

To compute the significance of the Pearson correlation between two time series, we used surrogate data generated by either permutation or the random phase procedure. Permutation surrogate time series were generated by randomly shuffling data. Random phase surrogate time series were generated by Ebisuzaki’s random phase method (Ebisuzaki, 1997) as implemented in the rEDM (version 1.5) function make_surrogate_data. For a pair of time series $\left[X_{1}\,\left(1\right),X_{1}\,\left(2\right),\ldots ,X_{1}\,\left(1000\right)\right],\left[X_{2}\,\left(1\right),X_{2}\,\left(2\right),\ldots ,X_{2}\,\left(1000\right)\right]$, we first computed the Pearson correlation $\hat{\rho }$ between the two time series. We then replaced the *X*_2_ values with surrogate time series and recomputed the Pearson correlation as $\tilde{\rho }$. We computed this shuffled correlation 9,999 times (permutation) or 499 times (random phase) to get a null distribution $\left[{\tilde{\rho }}_{1},{\tilde{\rho }}_{2},\ldots ,{\tilde{\rho }}_{n}\right]$. Following Schreiber and Schmitz, 2000, we computed the p value as

(5) $$p=\left(N_{s\,t\,r\,o\,n\,g\,e\,r}+1\right)/\left(N_{s\,u\,r\,r}+1\right)$$

where $N_{s\,u\,r\,r}$ is the number of surrogates, $N_{s\,t\,r\,o\,n\,g\,e\,r}$ is the number of surrogate correlations $\tilde{\rho }$ whose magnitude was greater than or equal to the magnitude of the original correlation $\hat{\rho }$, and the “+1” terms account for the original correlation $\hat{\rho }$.

#### Methodological details for Figure 4

The system of equations was numerically integrated using the ode45 method in Matlab from t=0 to t=200 in time steps of 0.03, and plotted in the delay space Z with τ=3.6. The initial condition for all state variables (V, W, X, Y, Z, $\frac{d\,X}{d\,t}$, $\frac{d\,Y}{d\,t}$, and $\frac{d\,V}{d\,t}$) was 1. For panel F, measurement noise was added to Y⁢(t). Specifically, noisy data were generated as:

$$Y^{obs}(t)∼Unif\left(Y(t)-3^{1/2}\left(0.15\Delta_{Y}\right),Y(t)+3^{1/2}\left(0.15\Delta_{Y}\right)\right)$$

where Unif⁢(a,b) is a uniform random variable bounded by a and b, and $\Delta_{Y}$ is the difference between the maximum and minimum values of Y⁢(t) between t=0 and t=200. These noise parameters are chosen so that $Y^{o\,b\,s}\,\left(t\right)$ is centered at Y⁢(t) and has a standard deviation of $0.15\,\Delta_{Y}$ .

#### Methodological details for Figure 5

The dynamics in the top row of Figure 5 were generated from the equations:

X⁢(t)=sin⁢(t)+0.5⁢t

$$Z\,\left(t\right)=0.1\,{\left(t-10\right)}^{2}$$

This continuous-time system was discretized from t=1 to t=20 on an evenly spaced grid of 400 data points for visualizing delay spaces where the time delay is 50 time points (i.e. τ=50⁢(20-1)/(400-1)).

The dynamics in the second row of Figure 5 were generated from the equations:

X⁢(t+1)=X⁢(t)⁢(3.61-3.61⁢X⁢(t))

Z(t+1)=Z(t)(3.61−3.61X(t))

with initial conditions of X⁢(1)=0.4 and Z⁢(1)=0.7. For this system, τ=1 and t=1,2,…,2000 were used to make the plots of delay spaces.

The dynamics in the third row of Figure 5 were generated from the equations:

$$\begin{array}{l} \frac{dX^{2}}{dt}=-X(t)\\ \frac{dZ^{2}}{dt}=-25Z(t) \end{array}$$

with initial conditions of $X\,\left(1\right)=X^{\prime }\,\left(1\right)=Z\,\left(1\right)=Z^{\prime }\,\left(1\right)=1$. For this system, τ=0.9 was used for delay spaces. This continuous-time system was numerically integrated using the ode45 method in Matlab from t=0 to t=13.998 on a grid of 4,667 evenly-spaced time points for plotting dynamics, and time points t=0.003 through t=7.698 were used for visualizing delay spaces.

The dynamics in the bottom row of Figure 5 were generated from the classic Lorenz attractor equations:

$$\begin{array}{l} \frac{dX}{dt}=-10X(t)+10Y(t)\\ \frac{dY}{dt}=28X(t)-Y(t)-X(t)Z(t)\\ \frac{dZ}{dt}=-\frac{8}{3}Z(t)+X(t)Y(t) \end{array}$$

with initial conditions of X⁢(0)=Y⁢(0)=Z⁢(0)=1. A delay of τ=0.14 was used to make delay spaces. This continuous-time system was numerically integrated using the ode45 method in Matlab from t=0 to t=399.98 on an evenly spaced grid of 5715 data points for visualizing delay spaces.

#### Methodological details for Figure 7

##### Ground truth model and data generation

We used the ground truth model:

$$\begin{array}{l} S_{1}(t+1)=max\left(0,S_{1}(t)\left(1.2-0.1S_{1}(t)+D_{1}(t)\right)+\epsilon_{p1}(t)\right)\\ S_{2}(t+1)=max\left(0,S_{2}(t)\left(1.1-0.2S_{2}(t)+D_{2}(t)+0.3S_{1}(t)\right)+2.5\epsilon_{p2}(t)\right) \end{array}$$

$S_{1}\,\left(t\right)$ and $S_{2}\,\left(t\right)$ represent the population sizes of species 1 and 2 at time *t*. and $D_{2}\,\left(t\right)$ are the values of periodic drivers at time t. Specifically, in both the two-driver and one-driver cases:

$$D_{1}\,\left(t\right)=0.05\,\sin\,\left(t+\phi_{1}\right)+0.05\,\sin\,\left(\frac{5\,t}{6}+\phi_{1}\right)$$

In the two-driver case:

$$D_{2}\,\left(t\right)=0.1\,\sin\,\left(\frac{t}{\sqrt{10}}+\phi_{2}\right)$$

Conversely, in the one-driver case $D_{2}\,\left(t\right)=0$. The process noise terms $\epsilon_{p\,1}\,\left(t\right)$ and $\epsilon_{p\,2}\,\left(t\right)$ are both IID normal random variables with mean of 0 and with shared standard deviation $\sigma_{p}$. Specifically, for any pair of times $t_{1}\neq t_{2}$, $\epsilon_{p\,1}\,\left(t_{1}\right)$ and $\epsilon_{p\,1}\,\left(t_{2}\right)$ are independent, and similarly for $\epsilon_{p\,2}$. Also, all values $\epsilon_{p\,1}\,\left(1\right),\epsilon_{p\,1}\,\left(2\right),\cdots$ are independent of all values $\epsilon_{p\,2}\,\left(1\right),\epsilon_{p\,2}\,\left(2\right),\cdots$. At the beginning of each replicate simulation, the phases $\phi_{1}$ and $\phi_{2}$ are independently assigned a random number from a uniform distribution between 0 and 2⁢π, and do not change with time.

To generate data without measurement noise, we simulated this system for t=1,2,…,400 with the initial conditions $S_{1}(1)=2;S_{2}(1)=4.5$. We used the final 200 time points for inference to help ensure that the system had reached equilibrium behavior.

We also introduced additive measurement noise to simulate instrument uncertainty:

$$\begin{array}{l} S_{1}^{obs}(t)=S_{1}(t)+\epsilon_{m1}(t)/1.5\\ S_{2}^{obs}(t)=S_{2}(t)+\epsilon_{m2}(t) \end{array}$$

where $S_{1}^{o\,b\,s}$ and $S_{2}^{o\,b\,s}$ represent the observed values (i.e. noisy measurements) of *S*_1_ and *S*_2_. $\epsilon_{m\,1}\,\left(t\right)$ and $\epsilon_{m\,2}\,\left(t\right)$ are also IID normal random variables with mean of 0 and standard deviation $\sigma_{m}$. The tables in Figure 7D are generated by varying $\sigma_{p}$ from 0 to 8 and varying $\sigma_{m}$ from 0 to 1.

#### Causal analysis using Granger causality and CCM

For each combination of $\sigma_{m}$ and $\sigma_{p}$ (the standard deviation of measurement noise and process noise, respectively), we generated 1000 time series for *S*_1_ and *S*_2_ as described above. For each replicate pair of time series, we used Granger causality and CCM to infer whether *S*_1_ causes *S*_2_ (it does) and whether *S*_2_ causes *S*_1_ (it does not).

#### Granger causality inference

We used the multivariate Granger causality Matlab package (MVGC, Barnett and Seth, 2014). We used the following settings:

- regmode = ’OLS’ (We fit the autoregressive model by the ordinary least squares method).
- icregmode = ’LWR’ (We determined the information criterion using the LWR algorithm. This is the default setting).
- morder = ’AIC’ (We used Akaike information criterion to determine the number of lags in the autoregressive model).
- momax = 50 (We used a maximum of 50 lags in the autoregressive model).
- tstat = ” (We used Granger’s F-test for statistical significance. This is the default setting).

We inferred the presence of a causal link if the p-value was less than or equal to 0.05. We inferred no causal link otherwise. When $\sigma_{m}$ and $\sigma_{p}$ were both 0, the MVGC package (correctly) exited with an error on most trials. We reported this as ‘unsuitable data’ in Figure 7D & E.

When $\sigma_{m}$ and $\sigma_{p}$ are both 0, the inferred spectral radius of the stochastic process is close to 1, and the MVGC routines can be prohibitively slow (i.e. when running 1,000 trials, the program would hang at an early stage for hours). In this case, the authors note that switching from the package’s default single-regression mode to an alternative dual-regression mode may improve runtime (Barnett and Seth, 2014). We thus switched to the dual-regression mode when the spectral radius was between 0.9999 and 1 (a spectral radius of 1 or more causes an error). This fix had no effect on benchmark results as long as at least one of $\sigma_{m}$ and $\sigma_{p}$ was not 0.

#### Convergent cross mapping

Convergent cross mapping looks for a delay map from X to Y. That is, CCM looks for a map from [X⁢(t),X⁢(t-τ),X⁢(t-2⁢τ),…,X⁢(t-(E-1)⁢τ)] to Y⁢(t). Thus in order to apply CCM one needs to choose the delay τ and the vector length (dimension of the delay space) *E.* The parameters E and τ should ideally be ‘generic’ in the sense of Takens’ theorem: we want to avoid line-crossing (such as the symbol ‘∞’) in the delay space, because otherwise, $\Phi^{-1}$ in Appendix 4—figure 2 does not exist. There are different ways to do this, but no method is obviously the best (Harnack et al., 2017; Cobey and Baskerville, 2016).

Following Cobey and Baskerville, 2016 and Sugihara et al., 2012 we chose τ and E to maximize univariate one-step-ahead forecast of the putative causee X. That is, for X⁢(n), we try to predict X⁢(n+1) using the simplex projection method by finding delay vectors in the training data of X that are most similar to [X⁢(n),X⁢(n-τ),X⁢(n-2⁢τ),…,X⁢(n-(E-1)⁢τ)], and take weighed average of their X values one step in the future (i.e. Figure 6A where X=Y and the prediction lag is 1). If the delay space has a line crossing, then at the cross-point, a one-step-ahead forecast may have more than one possible outcome and thus perform poorly. In more detail, we made one-step-ahead forecasts within the time range 201–400 (we did not use time range 1–200 to avoid transient dynamics). As per the field standard, we used leave-one-out cross-validation to do simplex projection. That is, when making a forecast for a time t, we used all times within 201–400 other than t as training data (200 time points). We performed a grid search, varying τ from 1 to 6 and varying E from 1 to 6. We then used the combination of τ and E that maximized the forecast skill (the Pearson correlation between forecasts and true values) for subsequent CCM analysis. Additionally, following (Sugihara et al., 2012), if the optimal combination of τ and E failed to give a significantly positive forecast skill, we did not report CCM results for that trial and reported the trial as “unsuitable data”. To test whether forecast skill is “significantly positive”, we ask whether it is robust to small changes in the training dataset. To do so, we used a naive bootstrap approach to create different versions of training libraries composed of randomly chosen delay vectors (sampling with replacement: some vectors may not be sampled and others may be sampled more than once) from the original training data using the ’random_libs’ setting in the rEDM (version 1.5) ccm method. The training library size (the number of delay vectors in the library) was chosen to be 200. We then calculated forecast skills with 300 such libraries and considered the forecast skill “significant” if at least least 95% gave a forecast skill greater than 0.

Having chosen τ and E, we checked three CCM criteria to infer causality (criteria 1–3 in Figure 6) using rEDM version 1.5. We did not use the fourth criterion (the prediction lag test) since its interpretation is unclear for periodic systems (Appendix 4—figure 5). For all three criteria, we used the same cross-validation setting that we used to choose τ and E. The first CCM criterion is that cross map skill is greater than 0. Thus, we computed cross map skill using the maximum possible number of distinct delay vectors (200-(E-1)⁢τ) and compared this value to 0.

The second CCM criterion is that the cross map skill from causee to causer with real data must be greater than the cross map skill when the putative causer is replaced with surrogate data. To test this criterion, we first computed cross map skill using the same training and testing time points as before to obtain a single cross map skill value. We then repeatedly (1000 times) computed cross map skill in the same way, but now with the putative causer time series replaced with random phase surrogate data. Random phase surrogate data were generated by Ebisuzaki’s method as implemented in the rEDM function make_surrogate_data. We then computed the p-value according to Equation 5. A putative causal link would pass this criterion if the p-value was less than or equal to 0.05.

The third CCM criterion is that cross map skill increases with more training data. Following Cobey and Baskerville, 2016, we again used a naive bootstrap approach to test for this criterion. Specifically, we computed the cross map skill with a training library composed of randomly chosen delay vectors sampled with replacement from the original training data time points. We used either a large library with 200-(E-1)⁢τ available training vectors as used previously, or a small library with 15 training vectors. For each of 1000 bootstrap trials, we compared the cross map skill from a randomly chosen small library to the cross map skill from a randomly chosen large library. We said that the cross map skill increased with training data if the cross map skill of the large library was greater than that of the small library in at least 95% of the 1000 bootstrap trials.

For ‘alternative’ CCM testing, we only changed how the third CCM criterion (cross map skill increases with more training data) were tested. Here, instead of using the bootstrap test of Cobey and Baskerville, 2016, we tested the third CCM criterion using Kendall’s τ test as suggested in Chang et al., 2017. To do this, we varied the library size from a minimum of 15 vectors to the the maximum library size (200-(E-1)⁢τ), in increments of 3 vectors. For each library size, we computed cross map skill using 50 libraries randomly sampled without replacement (e.g. the 50 libraries would be identical at the maximal library size). We then computed the median cross map skill for each library size. Finally we ran a 1-tailed Kendall’s τ test for a positive association between library size and median cross map skill. We used the function stats.kendalltau from the Python package SciPy to compute a 2-tailed p-value, and then divided this p-value by two to get a 1-tailed p-value. We said that cross map skill increased with training data if the τ statistic was positive and the 1-tailed p-value was ≤0.05.

#### Methodological details for Appendix 1—figure 3

The original subpopulation distributions are normal distributions with standard deviation of 10 and mean of 100 (male) or 130 (female). Each sampling plot shows 300 random samples.

#### Methodological details for Appendix 4—figure 2

To generate data for panels C-J, the system of panel A was numerically integrated using the ode45 method in Matlab with a time step of 0.005 and with the initial condition that X, Y, Z, $\frac{d\,X}{d\,t}$, $\frac{d\,Y}{d\,t}$ were all set to one at t=0. Panels C, E, F, G, and I show data from a single period. For panel H the system was integrated for about five periods to more clearly visualize the lack of a continuous delay map. For panel J, the system was integrated for 1 period for the main panel and about 12 periods (to increase the sampling density) for the inset. This allows us to better see the separated legs of the curve upon zooming in. Panels C, D, F, H, and J were colored mod⁢(t,T). That is, they were colored by the remainder of t (time) after dividing by T (here T=2⁢π). τ=3.6 was used for all delay spaces.

#### Methodological details for Appendix 4—figure 3

All systems were discretized from t=1 to t=20 on an evenly spaced grid of 200 points for visualizing delay spaces.

The dynamics in the top row were generated from the equations:

X⁢(t)=sin⁢(t)+0.5⁢t

Y⁢(t)=sin⁢(1.3⁢t)

A delay time of 12 time indices (i.e. τ=12⁢(20-1)/(200-1)) was used for constructing delay spaces.

The dynamics in the second row were generated from the equations:

X⁢(t)=sin⁢(1.3⁢t)

Y(t)=Y(t−δ)(3.77−3.77∗Y(t−δ))

with δ=(20-1)/(200-1) and the initial condition Y⁢(1)=0.3. A delay time of 25 time indices (i.e. τ=25⁢(20-1)/(200-1)) was used for constructing delay spaces.

In the third row the dynamics are identical to the first row, except that X and Y are switched, and τ=25 time indices was used for constructing delay spaces.

#### Methodological details for Appendix 4—figure 4

Top row: For this system, we used the initial conditions $W\,\left(0\right)=\dot{W}\,\left(0\right)=X\,\left(0\right)=Y\,\left(0\right)=\dot{Y}\,\left(0\right)=1$. We numerically integrated this system using ode45 in Matlab with a time step of 0.03. We composed delay vectors of length E=3 with a delay of τ=3.6. We visualized the delay space using data from t=0 through t=29.97 (time indices 1–1000). For CCM with temporally separate training and testing sets, we used data from t=0 through t=14.97 (time indices 1–500) for training data and data from t=15 through t=29.97 (time indices 501–1000) for testing. Specifically, in the rEDM (version 0.7.2) ccm method we set the “lib” argument to “c(1, 500)” and set the “pred” argument to “c(501, 1000)”. We used rEDM version 0.7.2 for this analysis because we found that it more easily produced distinct training and test sets than later versions (on a computer running MacOS 11.6 and R version 4.0.2). For CCM with temporally interspersed training and testing sets, we set both the lib and pred arguments to “c(1,1000)”. This setting instructs rEDM to use leave-one-out cross-validation.

Second row: Ground truth data generation and analysis were the same as in the top row, except that the roles of X and Y were swapped.

Third row: For this system, we used the initial conditions $W\,\left(0\right)=0,\dot{W}\,\left(0\right)=1/1.6,X\,\left(0\right)=1.3,Y\,\left(0\right)=1.5$. We numerically integrated this system using ode45 in Matlab with a time step of 0.1. We visualized the delay space using data from t=0 through t=40 (time indices 1–401). We used the delay vector parameters (E=3,τ=3.0). For CCM with temporally separate training and testing sets, we used data from t=0 through t=19.9 (time indices 1–200) for training data and data from t=20 through t=39.9 (time indices 201–400) for testing. For CCM with temporally interspersed training and testing sets, we used cross-validation over the entire range t=0 through t=39.9.

Bottom row: We discretized this system with a time step of 0.05. We visualized the delay space using data from t=0 through t=20 (time indices 1–401). We used the delay vector parameters (E=2,τ=2.5). For CCM with temporally separate training and testing sets, we used data from t=0 through t=9.95 (time indices 1–200) for training data and data from t=10 through t=19.95 (time indices 201–400) for testing. For CCM with temporally interspersed training and testing sets, we used cross-validation over the entire range t=0 through t=19.95.

For convergent cross mapping, we used the same τ and E as for visualizing delay spaces (see above). “Training data size” on the horizontal axis is the number of delay vectors in the training library. Each dot in these CCM plots represents the average forecast skill over 300 randomly chosen libraries. Error bars represent the 95% confidence interval as calculated by the bias-corrected and accelerated bootstrap (1,000 bootstraps) as implemented in Matlab’s bootci function. Error bars are the same color as the dots and so are not visible when they fit inside the dots.

#### Methodological details for Appendix 4—figure 5

Top row: For this system, we used the initial conditions X⁢(1)=0.2,Y⁢(1)=0.4 and composed delay vectors of length E=2 with a delay of τ=2. We visualized the delay space using data from time points 501–2000. We used points 801–1000 for training data and points 1001–2000 for testing cross map predictions.

Second row: For this system, we used the initial conditions X⁢(1)=0.4,Y⁢(1)=0.2 and the delay vector parameters (E=2,τ=1). We visualized the delay space using data from time points 501–2000. We used points 801–1000 for training data and points 1001–2000 for testing cross map predictions.

Third row: For this system, we used the initial conditions X⁢(1)=0.2,Y⁢(1)=0.4 and the delay vector parameters (E=3,τ=2). We visualized the delay space using data from time points 501–2000 (time points 1-$6\times 10^{5}$ for the zoomed-in inset). We used points 801–1000 for training data and points 1001–2000 for testing cross map predictions.

Fourth row: For this system, we used the initial conditions W⁢(0)=Y⁢(0)=0 and $X\,\left(0\right)=\dot{W}\,\left(0\right)=\dot{Y}\,\left(0\right)=1$. We numerically integrated this system using ode45 in Matlab with a time step of 0.1. We visualized the delay space using data from t=50.1 through t=200 (time indices 501–2000). We used the delay vector parameters (E=3,τ=7.2). We used data from t=70.1 through t=100 (time indices 701–1000) for training data and data from t=100.1 through t=200 (time indices 1001–2000) for testing cross map predictions.

Fifth row: For this system, we used the initial conditions X⁢(1)=0.2,Y⁢(1)=0 and composed delay vectors of length E=2 with a delay of τ=2. We visualized the delay space using data from time points 501–2000. We used points 801–1000 for training data and points 1001–2000 for testing cross map predictions.

Sixth row: For this system, the “initial” conditions specified the first three time points since we included a lag of 3. Thus, for k=1,2,3, W⁢(k)=0.2, X⁢(k)=0.4, and Y⁢(k)=0.3. We composed delay vectors of length E=3 with a delay of τ=1. We visualized the delay space using data from time points 501–2000. We used points 801–1000 for training data and points 1001–2000 for testing cross map predictions.

For convergent cross mapping (in rEDM version 0.7.2), we used the same τ and E as for visualizing delay spaces. The training data size is the number of delay vectors in the training library. For the plots in the fourth column, we chose 300 random libraries of training delay vectors with variable training data size, and used the standard prediction lag of 0. Delay vectors were chosen without replacement. Note that at large training data size, some or all of the 300 random libraries can be identical. Each dot in these CCM plots represents the average forecast skill over all 300 randomly-chosen libraries. Error bars represent the 95% confidence interval as calculated by the bias-corrected and accelerated bootstrap (1,000 bootstraps) as implemented in Matlab’s bootci function. Error bars are the same color as the dots and so are not visible when they fit inside the dots.

In all rows, the cross map skill for the putative causer Y was greater than for at least 95% of random phase surrogate time series (purple dot). The 5% cutoff value was computed for the maximum library size (156 for row four and ∼200 for all other rows) by running the CCM procedure after replacing the putative causer Y with 500 random phase surrogate time series generated using the rEDM function make_surrogate_data.

For the plots in the fifth column we used the full library contained within the training data window (156 delay vectors for row four and ∼200 for all other rows) and varied the prediction lag. We did not use random libraries for these plots.

#### Methodological details for Appendix 4—figure 6

To generate randomized parameter sets, we randomly selected *R*_*X*_, *R*_*Y*_, $A_{X\,Y}$ and $A_{Y\,X}$ from uniform distributions. We also randomly selected the initial conditions X⁢(1) and Y⁢(1) from uniform distributions. To make systems in the ‘friendly’ parameter regime, we drew *R*_*X*_ and *R*_*Y*_ independently from the range 3.7–3.9, we drew $A_{X\,Y}$ and $A_{Y\,X}$ independently from the range 0.05–0.1, and we drew X⁢(1) and Y⁢(1) independently from the range 0.01–0.99. These are the same parameters used in the randomized numerical simulations of Ye et al., 2015. Next, to make systems in the ‘pathological’ parameter regime, we drew *R*_*X*_ from the range 3.7–3.9, we drew *R*_*Y*_ from the range 3.1–3.3, we drew $A_{X\,Y}$ and $A_{Y\,X}$ independently from the range 0.15–0.2, and we drew X⁢(1) and Y⁢(1) independently from the range 0.01–0.99. For both parameter regimes we randomly chose 250 sets of parameters and ran the system for 3,000 time points. Occasionally a randomly chosen system would leave the basin of attraction and reach large values, represented on the computer as positive or negative infinity, or ‘not a number’. When this occurred, we discarded the data and resampled parameters.

To apply CCM (in rEDM version 0.7.2) on each system, we generated a training library of delay vectors of X by randomly selecting 200 vectors from among time points 100–2000. We then evaluated cross map skill from delay vectors of X to values of Y at points 2001–3000. Following Ye et al., 2015, we used delay vectors of length E=2 and a delay duration of τ=1. We evaluated cross map skill with a prediction horizon of -8 through 8.
